# Systemic RNA Interference Defective (SID) genes modulate dopaminergic neurodegeneration in *C. elegans*

**DOI:** 10.1371/journal.pgen.1010115

**Published:** 2022-08-19

**Authors:** Anthony L. Gaeta, J. Brucker Nourse, Karolina Willicott, Luke E. McKay, Candice M. Keogh, Kylie Peter, Shannon N. Russell, Shusei Hamamichi, Laura A. Berkowitz, Kim A. Caldwell, Guy A. Caldwell

**Affiliations:** 1 Department of Biological Sciences, The University of Alabama, Tuscaloosa, Alabama, United States of America; 2 Center for Convergent Bioscience and Medicine, The University of Alabama, Tuscaloosa, Alabama, United States of America; 3 Alabama Research Institute on Aging, The University of Alabama, Tuscaloosa, Alabama, United States of America; 4 Departments of Neurology and Neurobiology, Center for Neurodegeneration and Experimental Therapeutics, Nathan Shock Center of Excellence for Basic Research in the Biology of Aging, University of Alabama at Birmingham, Heersink School of Medicine, Birmingham, Alabama, United States of America; Brown University, UNITED STATES

## Abstract

The fine-tuning of gene expression is critical for all cellular processes; aberrations in this activity can lead to pathology, and conversely, resilience. As their role in coordinating organismal responses to both internal and external factors have increasingly come into focus, small non-coding RNAs have emerged as an essential component to disease etiology. Using Systemic RNA interference Defective (SID) mutants of the nematode *Caenorhabditis elegans*, deficient in gene silencing, we examined the potential consequences of dysfunctional epigenomic regulation in the context of Parkinson’s disease (PD). Specifically, the loss of either the *sid-1* or *sid-3* genes, which encode a dsRNA transporter and an endocytic regulatory non-receptor tyrosine kinase, respectively, conferred neuroprotection to dopaminergic (DA) neurons in an established transgenic *C*. *elegans* strain wherein overexpression of human α-synuclein (α-syn) from a chromosomally integrated multicopy transgene causes neurodegeneration. We further show that knockout of a specific microRNA, *mir-2*, attenuates α-syn neurotoxicity; suggesting that the native targets of *mir-2*-dependent gene silencing represent putative neuroprotective modulators. In support of this, we demonstrated that RNAi knockdown of multiple *mir-2* targets enhanced α-syn-induced DA neurodegeneration. Moreover, we demonstrate that *mir-2* overexpression originating in the intestine can induce neurodegeneration of DA neurons, an effect that was reversed by pharmacological inhibition of SID-3 activity. Interestingly, *sid-1* mutants retained *mir-2*-induced enhancement of neurodegeneration. Transcriptomic analysis of α-syn animals with and without a *sid-1* mutation revealed 27 differentially expressed genes with human orthologs related to a variety of diseases, including PD. Among these was *pgp-8*, encoding a P-glycoprotein-related ABC transporter. Notably, *sid-1*; *pgp-8* double mutants abolished the neurodegeneration resulting from intestinal *mir-2* overexpression. This research positions known regulators of small RNA-dependent gene silencing within a framework that facilitates mechanistic evaluation of epigenetic responses to exogenous and endogenous factors influencing DA neurodegeneration, revealing a path toward new targets for therapeutic intervention of PD.

## Author summary

The progressive death of neurons that produce the neurotransmitter dopamine is a clinical hallmark of Parkinson’s disease (PD). An integrated response to environmental and genetic factors leads to expression changes in specific genes and non-protein-coding regulatory molecules called dsRNAs that influence the pathology underlying PD. Here we report, for the first time in an animal model of PD, that mutations in genes encoding proteins which function in the cellular import of dsRNA protect dopamine neurons from degeneration. By generating a profile of individual genes affected when dsRNA transport is incapacitated, we established a foundation for the systematic analysis of their distinct contributions to neuroprotection. One subclass of dsRNAs, termed microRNAs, function analogously to a conductor of an orchestra by controlling hundreds of genes, simultaneously, to coordinate cellular processes. We identified a single microRNA that suppresses the activity of numerous genes in dopamine neurons and contributes to neurodegeneration. Genomic knockout of this deleterious microRNA, or prevention of its transport into dopamine neurons, unmasked a set of previously unreported neuroprotective proteins. This study supports a hypothesis whereby mechanisms that “fine tune” dopamine availability intersect with regulators of dsRNA transport to cooperatively maintain an optimal balance between neuronal activity, neurodegeneration, and survival.

## Introduction

The silencing of genes is a fundamental mechanism by which cells can dynamically regulate gene expression to meet the specific needs of an organism more precisely [1–3]. Critical to organismal health, and potentially affecting all aspects of ecological fitness, it is no surprise that a multitude of ways exist for cells to execute this process. These include utilizing small, double-stranded RNAs (dsRNAs) such as microRNAs (miRNAs), piwi-interacting RNAs (piRNAs), and small interfering RNAs (siRNAs), to alter the expression of genes or change the epigenetic landscape of DNA that can indirectly lead to differential gene expression [4–6]. Gene silencing by dsRNAs can be accomplished by either full or partial binding to messenger RNA (mRNA) transcripts, subsequently suppressing or completely inhibiting the translation of the transcript into protein [2,7]. Mechanisms for endogenous expression and transport of small dsRNAs in the coordinated regulation of gene expression, have evolved across species that facilitate a rapid, dynamic, and adaptive means of organismal response to internal or external conditions [6,8]. Researchers have also learned to exploit the exquisite specificity of this mechanism, using RNA interference (RNAi) to deplete target RNA transcripts and determine the functional implications of knocking down the activity of distinct genes. In this manner, a greater understanding of the respective impact that individual genes have as contributors to general organismal health and specific phenotypes, mechanisms, or pathways, has been substantially expedited through the application of RNAi [9–13]. Its revolutionary role in expanding our functional genomic toolkit aside, significant gaps remain in our basic understanding of RNAi and how gene silencing is effectuated through the organismal transmission of dsRNAs.

Small RNA-dependent changes in gene expression have been shown to contribute not only to beneficial processes such as stress responses, but also to pathology, where they have been revealed to be key factors in these processes [14–18]. The genome of the nematode *Caenorhabditis elegans* possesses a class of genes including *sid-1* and *sid-3*, designated Systemic RNA Interference Defective (SID), that encode proteins necessary for the organismal distribution and cellular transport of dsRNAs to trigger the selective targeting of transcripts for silencing [19,20]. SID-1 is a transmembrane transporter protein that imports dsRNA into various cell types, facilitating RNA-mediated gene silencing [20–22]. Mutant *sid-1* animals are phenotypically characterized as being resistant to RNAi and lack the capacity to confer the systemic spread of dsRNA across the cellular boundaries and tissues of this metazoan nematode [21]. SID-3 is a non-receptor tyrosine kinase that resides in the cytoplasm that exhibits binding activity to clathrin heavy chains and prevents clathrin-dependent endocytosis, ultimately inhibiting the internalization of plasma membrane-localized proteins [23,24]. In this manner, the mammalian ortholog of SID-3, termed ACK1, functions as a form of “brake” on endocytosis, which when released leads to the endocytic recycling of plasma membrane proteins. In DA neurons, ACK1 has been shown to regulate the density of the dopamine transporter (DAT) protein on neuronal membranes, thereby modulating DA reuptake from the synapse [25]. In an analogous scenario, *C*. *elegans* SID-3 is required for the efficient import of dsRNA into cells, as *sid-3* mutants exhibit a significant reduction in the capacity for environmental RNAi [23]. We posit that this is due to the involvement of SID-3 in maintaining baseline levels of SID-1 on cell surfaces, parallel to the activity of ACK1 in modulating DAT internalization.

In this study, transgenic *C*. *elegans* overexpressing a hallmark pathological gene product of Parkinson disease (PD), human α-synuclein (α-syn), are used to determine the effects of alterations in the systemic dsRNA uptake machinery on neuronal health and survival in the context of PD. Loss-of-function (*lof*) mutations in either *sid-1* or *sid-3*, were both found to protect DA neurons from the neurotoxic effects of transgenic multicopy α-syn overexpression in an established *C*. *elegans* model for the quantitative evaluation of DA neurodegeneration.

The collective outcomes of this research support a hypothesis that asserts factors influencing the systemic transport and cellular translocation of small dsRNAs represent previously undefined neuromodulators of DA neuron survival in the presence of proteotoxic stress resulting from α-syn overexpression in *C*. *elegans*. We examine this hypothesis through transcriptomic profiling of differential gene expression in *sid-1* mutants, as well through a combination of mutant analysis and selective overexpression of a specific *C*. *elegans* miRNA, termed *mir-2*, that modulates progressive α-syn-induced DA neurodegeneration, as observed in PD. We report results of a systematic analysis of evolutionarily conserved and functionally validated targets of *mir-2* regulation for their contribution to a state of heightened neuroprotection that is observed in the absence of *mir-2-*dependent transcriptional repression. These experiments add support for prior associative genetic relationships with PD and reveal new targets of potential therapeutic significance. Additionally, we utilize tissue-specific transgene expression to evaluate miRNA-mediated, cell non-autonomous effects on the DA neurodegeneration induced by α-syn in defined genetic mutant backgrounds to establish relationships between specific transport proteins as functional gatekeepers of dsRNA-dependent gene silencing. Taken together, this research advances our mechanistic understanding of epigenetic factors influencing organismal dynamics that effectively alter the intrinsic threshold of neuroprotection to the proteotoxic dysregulation underlying PD.

## Results

### *C*. *elegans sid-1* and *sid-3* mutants exhibit reduced DA neurodegeneration in a transgenic α-synuclein model of PD

**Fig 1:**
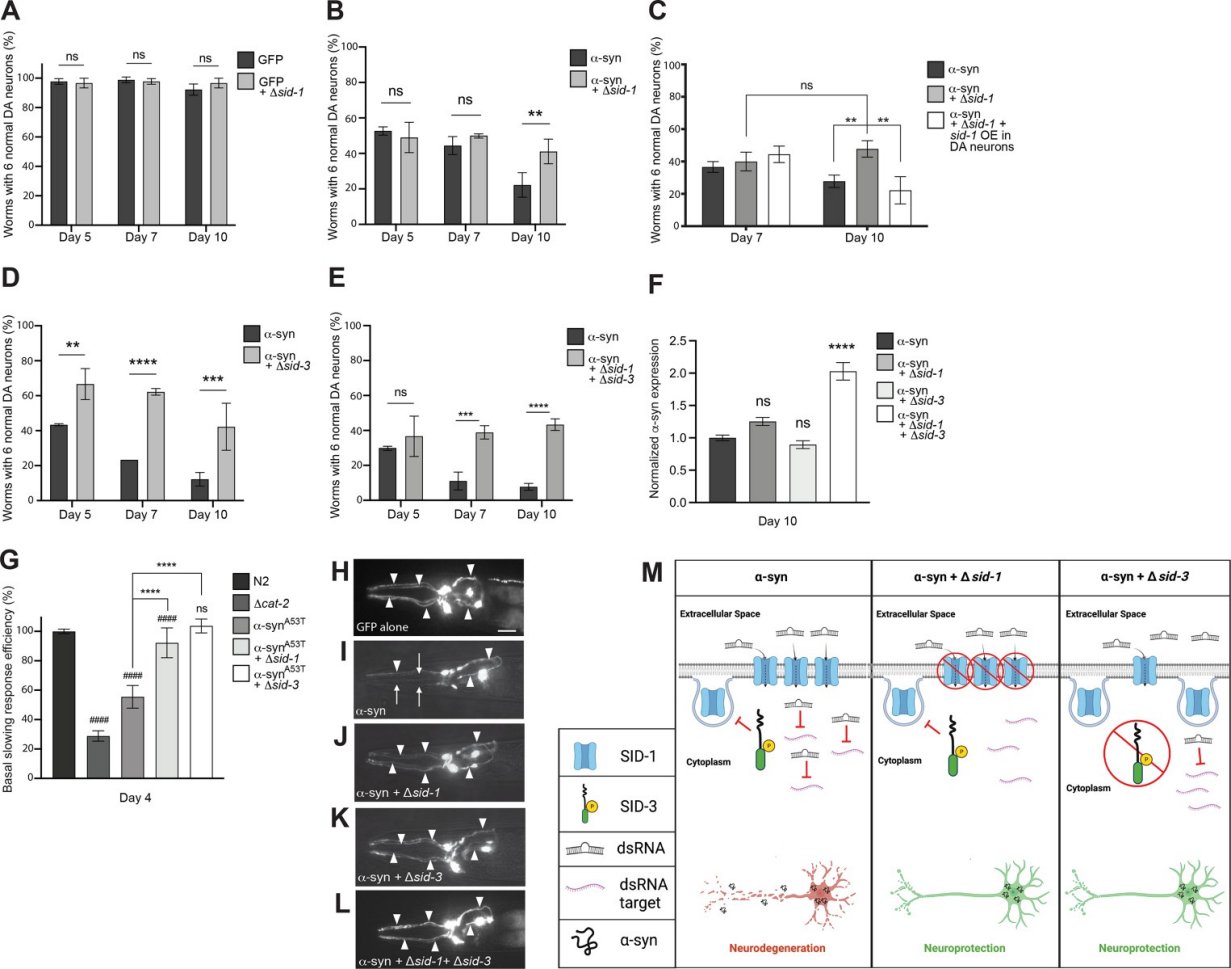
Loss of SID-1 and/or SID-3 functionality decreases DA neurodegeneration and improves DA neuron function. (**A-E**) DA neurons were scored for degeneration on days post-hatching indicated on X axes. Values represent mean + S.D. (n = 30 worms per genotype per replicate, 3 independent replicates per experiment). Two-Way ANOVAs with Šídák’s *post hoc* analyses were used for comparisons of transgenic animals at each time point (**A, B, D, E**) while a Tukey’s *post hoc* analysis was used to compare all conditions to each other in panel **C**; ns P ≥ 0.05, ** P < 0.01, *** P <0.001, **** P < 0.0001. (**F**) Expression levels of α-syn in α-syn only worms and α-syn worms with *sid* mutations. Expression is normalized to the α-syn only group. Total RNA was isolated at day 10 post-hatching. One-Way ANOVA with Dunnett’s *post hoc* analysis was used to compare α-syn alone to α-syn + *sid-1(pk3321)*, α-syn + *sid-3(ok973)*, and α-syn + *sid-1(pk3321)* + *sid-3(ok973)*; ns P ≥ 0.05, **** P < 0.0001. (**G**) Basal Slowing Response Efficiency assay, normalized to N2. *cat-2* mutants synthesize significantly less DA than N2 worms and serve as a positive control. Transgenic worms expressing a familial mutant of α-syn, without GFP in the neurons, P_*dat-1*_:: α-syn^A53T^, along with *sid-1(pk3321)* and *sid-3(ok973)* mutant backgrounds, were used in this assay to quantify early changes in neurotransmission. Worms were tested on day 4 post-hatching. One-Way ANOVA with Tukey’s *post hoc* analysis was used to compare all conditions to each other. Symbols above bars are in comparison to N2 control; ns P ≥ 0.05, #### P < 0.0001. Symbols above brackets are in comparison to bars indicated. **** P < 0.0001. (**H-L**) These images represent the six anterior DA neurons in characteristic worms expressing GFP in the six head neurons on day 10 post-hatching. Arrowheads indicate intact DA neurons while arrows indicate degenerated DA neurons. Scale bar, 20 μm. (**H**) This animal expresses GFP only and displays a full complement of 6 anterior DA neurons. (**I**) GFP + α-syn expressed in DA neurons where the arrowheads indicate only 3 neurons are intact while 3 neurons are degenerating (arrows). (**J**) GFP + α-syn expressed in DA neurons in a *sid-1(pk3321)* mutant background; all 6 anterior DA neurons are intact. (**K**) GFP + α-syn expressed in DA neurons in a *sid-3(ok973)* mutant background; all 6 anterior DA neurons are intact. (**L**) GFP + α-syn expressed in a *sid-1(pk3321); sid-3(ok973)* double mutant background; all 6 anterior DA neurons are intact. (**M**) A tripartite illustration (created with Biorender.com) depicting an interpretation of the results obtained where the activity of SID-1 and/or SID-3 modulates dsRNA import, leading to differential effects on α-syn-induced DA neurodegeneration. The left pane displays a scenario where α-syn is expressed in DA neurons, while *sid-1* and *sid-3* are both wildtype: activated SID-3 functions as an endocytic brake, thereby preventing endocytosis of SID-1, which maintains the transport of dsRNAs that silence target genes in DA neurons, rendering them vulnerable to neurodegeneration. The middle pane displays the situation when α-syn is expressed in DA neurons of animals in which *sid-1* is mutant and *sid-3* is wildtype: here, the loss of SID-1 function results in reduced dsRNA transport, thereby leading to the neuroprotection from α-syn overexpression observed, as a logical consequence of attenuated gene silencing and the concomitant transcriptional upregulation of protective gene products. In the right pane, *sid-1* is wildtype and *sid-3* is mutant in the same neurotoxic α-syn background: when SID-3 is incapacitated by mutation the endocytosis of SID-1 is no longer blocked, resulting in diminished dsRNA import and a reduction in the silencing of neuronal target gene expression that leads to the enhanced neuroprotection observed experimentally.

Evidence suggests that SID-1 localization is regulated by SID-3 [23], which putatively acts to maintain SID-1 at plasma membranes and facilitate silencing of target genes via dsRNAs imported by SID-1. To determine if endogenous gene silencing modulates DA neurodegeneration on its own, worms overexpressing GFP under the control of the DA neuron-specific *dat-1* promoter were crossed to *sid-1* mutants. When solely GFP is expressed in the DA neurons, a reproducibly negligible amount of neurodegeneration is observed (Fig 1H), whereas co-expression with α-syn results in progressive degeneration of DA neurons that worsens with age (Fig 1I). The *sid-1(pk3321)* missense mutant has been shown to be defective in the transmission of dsRNA between cells and tissues [21]. The *sid-1* mutation, and thus a loss of gene silencing ability induced by dsRNAs originating outside of cells, was found to have no significant effect on DA neurodegeneration at days 5, 7, and 10 post-hatching (Fig 1A). To determine if endogenous gene silencing functions in modulating α-syn-mediated DA neurodegeneration, transgenic nematodes overexpressing both GFP and non-mutant (“wildtype”) human α-syn exclusively in DA neurons were crossed to either *sid-1* or *sid-3* mutants. The *sid-3 (ok973)* allele represents a deletion of 1,330 base pairs and an insertion of 12 base pairs, deleting exons 11 and 12, and part of exon 13 [26]. Animals with the *sid-1* mutation in the α-syn background did not modulate neurodegeneration at day 5 or day 7 post-hatching, but significantly reduced neurodegeneration in older animals, specifically at day 10 post-hatching, when compared to the α-syn background alone (Fig 1B and 1J). This revealed an age-associated benefit conferred by *sid-1* mutants for DA neurons reflected by an increased capacity to resist the cellular stress posed by multicopy α-syn gene expression. In a reciprocal experiment, the neuroprotection bestowed by the *sid-1* mutation at day 10 post-hatching is lost when SID-1 activity is restored via selective *sid-1* overexpression in the DA neurons (Fig 1C). Additionally, the *sid-3* mutation in the α-syn background significantly reduced neurodegeneration at all ages examined (days 5, 7, and 10 post-hatching) (Fig 1D and 1K). When both *sid-1* and *sid-3* are mutant in the α-syn background, no significant change in neurodegeneration was observed at day 5 post-hatching, a time-point when *sid-3* mutant animals independently exhibited significant neuroprotection, and when the *sid-1* mutation alone displayed no significant change in DA neurodegeneration (Fig 1E). However, the *sid-1*; *sid-3* double mutants exhibited robust neuroprotection at days 7 and 10 post-hatching (Fig 1E and 1L), displaying a phenotype similar to worms with only *sid-3* mutation at these later time points. Thus, elimination or reduction of these established regulators of endogenous gene silencing leads to protection in an α-syn model of DA neurodegeneration (Fig 1M).

Although the loss of SID-1 and/or SID-3 functionality suggests that a decrease in endogenous gene silencing is responsible for the neuroprotection seen in either *sid-1* or *sid-3*, and in *sid-1*; *sid-3* double mutants, an alternative possibility remains that these mutations are decreasing the transcript levels of α-syn, and in this way leading to neuroprotection. To address this, RT-qPCR was performed on these *sid* mutant strains in the α-syn background at day 10 post-hatching, a time point at which all the strains exhibited neuroprotection. Animals with either *sid-1* or *sid-3* mutations in the α-syn background did not exhibit significantly altered α-syn transcript levels compared to α-syn in the wildtype background (Fig 1F). Interestingly, the *sid-1*; *sid-3* double mutants expressed significantly increased α-syn transcript levels compared to α-syn alone (Fig 1F). The root cause of an increase α-syn in only the double-mutant is unclear and warrants further investigation. Nevertheless, these data provide evidence that the loss of endogenous dsRNA-mediated gene silencing in the absence of SID protein function, and not transcriptional suppression of the α-syn transgene, at least partly underlies the observed neuroprotection.

### *sid-1* and *sid-3* mutants exhibit an improved neurobehavioral response in a transgenic *C*. *elegans* model of familial PD

Next, we wanted to determine if the reduced neurodegeneration observed in *sid-1* and *sid-3* mutant animals was also reflected by improved DA neurotransmission. To test this, Basal Slowing Response (BSR) assays were performed on *sid-1* and *sid-3* mutant animals. The BSR is a mechanosensory behavioral readout for DA neuron signaling and health in *C*. *elegans* and is dependent on the normal functionality of DA neurons [27]. BSR defects have been shown to precede neurodegeneration in α-syn-induced neurodegeneration models [28]. This behavioral assay can identify subtle defects in neurotransmission before neurodegeneration is visible via microscopical analysis. Since an A53T mutation in α-syn increases the rate of its oligomerization compared to WT α-syn, and leads to an early-onset, familial form of PD [29,30], worms overexpressing α-syn with the A53T mutation solely in the DA neurons (α-syn^A53T^) were used for this assay. Wildtype (N2) and *cat-2* mutant (DA deficient; tyrosine hydroxylase mutant) worms were used as controls to validate the assay, as the BSR is normal in N2 animals whereas *cat-2* mutant worms exhibit a defective BSR [27]. The α-syn^A53T^ worms also displayed a defective BSR compared to N2 controls, as expected (Fig 1G). Worms harboring either the *sid-1* or *sid-3* mutation in the α-syn^A53T^ background displayed a significantly improved BSR efficiency compared to the α-syn^A53T^ worms alone (Fig 1G). The *sid-3* mutation rescued the BSR to such a degree that it was indistinguishable from N2 animals (Fig 1G). Taken together with their impact on neurodegeneration, these behavioral data support the notion that decreases in SID-1 and SID-3 function robustly protect DA neurons from the dysfunction and neurotoxicity associated with excess α-syn, implying that loss and/or a reduction of endogenous RNA-mediated gene silencing is beneficial, in this specific context.

### Pharmacological inhibition of SID-3 diminishes the efficacy of exogenous RNAi and impacts DA neurodegeneration

**Fig 2:**
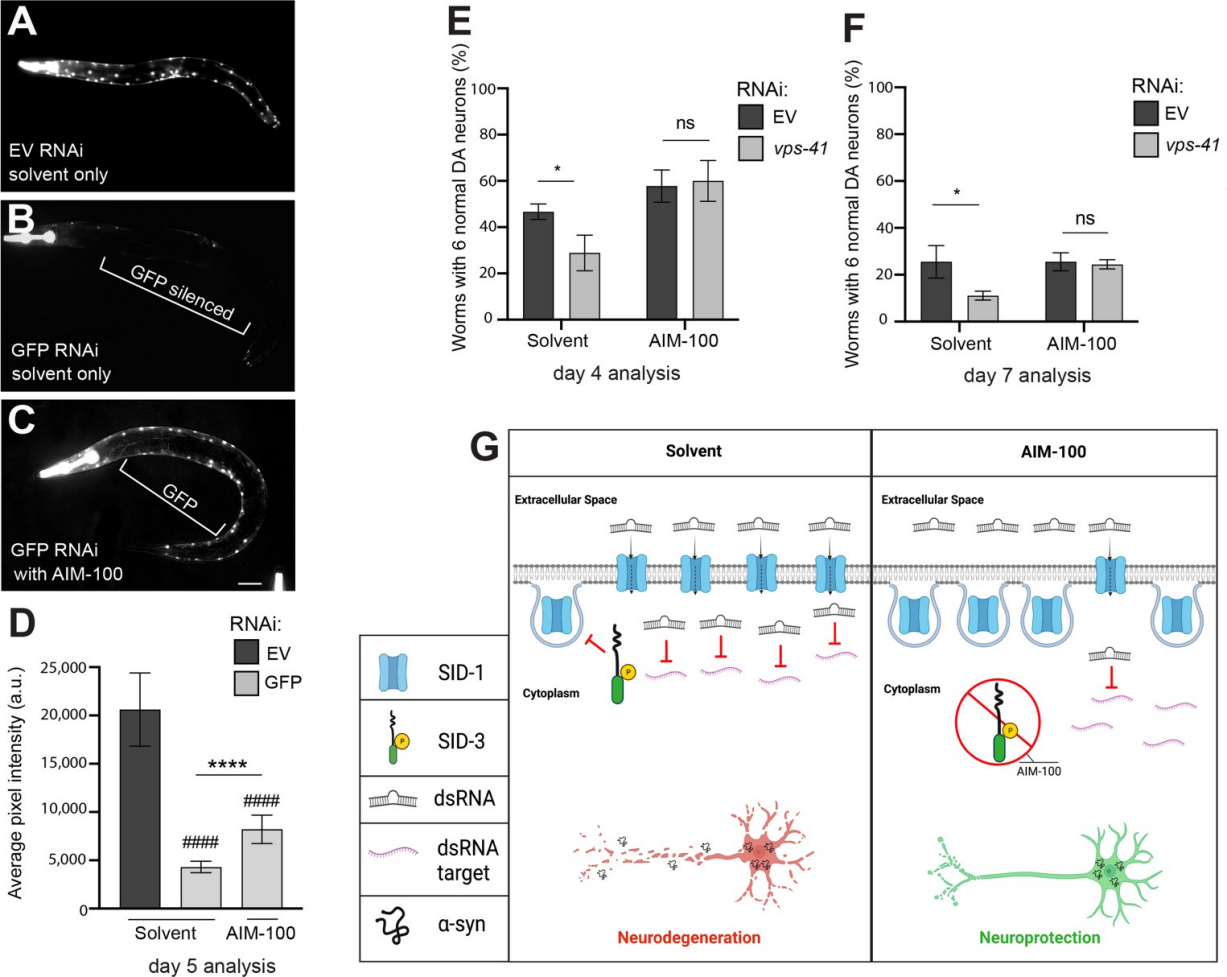
AIM-100 reduces dsRNA silencing in body wall muscle and decreases DA neurodegeneration. (**A-D**) AIM-100 diminishes RNAi silencing in body wall muscle cells. RNAi in representative worms overexpressing GFP in body wall muscle cells, with and without the SID-3 inhibitor AIM-100 (100 μM) in 0.1% ethanol solvent. Worms were tested on day 5 post-hatching. (**A**) Empty vector (EV) RNAi in worms overexpressing GFP in body wall muscle cells exposed to the solvent. (**B**) GFP RNAi in worms overexpressing GFP in body wall muscle cells exposed to the solvent. (**C**) GFP RNAi in worms overexpressing GFP in body wall muscle cells and administered AIM-100. Scale bar, 50 μm. **(D)** Average pixel intensity was measured at day 5 post-hatching in worms in the same conditions outlined in **(A-C)**. Values represent mean + S.D. (n = 30 worms per group per replicate, 3 independent replicates). Two-Way ANOVA with Tukey’s *post hoc* analysis was used to compare all groups to each other; Symbols above bars are in comparison to the solvent group with EV RNAi; #### P < 0.0001. Symbols above brackets are in comparison to bars indicated. **** P < 0.0001. (**E, F**) DA neurodegeneration levels in an RNAi-sensitive α-syn model background designed to allow knockdown of genes solely in DA neurons (strain UA196) and analyzed at days 4 and 7 post-hatching. Worms were either exposed to the 0.1% ethanol solvent control or AIM-100 (100 μM), and within each of these conditions, mock RNAi (empty vector) or *vps-41* (positive control) RNAi was performed. Values represent mean + S.D. (n = 30 worms per genotype per replicate, 3 independent replicates). Two-way ANOVA with Tukey’s *post hoc* analysis was used to compare all conditions to each other; ns P ≥ 0.05, * P < 0.05. Values represent mean + S.D. (n = 30 worms per genotype per replicate, 3 independent replicates). (**G**) An illustration (created with Biorender.com) depicting model scenarios for the import of dsRNA (targeting *vps-41*, an established control for DA neurodegeneration when knocked down) into DA neurons via SID-1 in transgenic animals expressing α-syn, either in the presence (left pane) or absence (right pane) of treatment with the selective inhibitor of SID-3 activity, AIM-100. The left pane represents SID-3 actively blocking the endocytosis of SID-1 in the absence of AIM-100 (solvent only), therefore maintaining dsRNA transport for the silencing of target genes, such as *vps-41*, which enhances DA neurodegeneration when knocked down. In contrast, the right pane displays a scenario that explains the observed inhibition of SID-3 by AIM-100 treatment seen when α-syn is expressed in DA neurons; in this scenario AIM-100 reduces the activity of SID-3 and allows for endocytosis of SID-1, thereby resulting in less SID-1 on cell surfaces and reduced dsRNA import into cells. This leads to the stability of endogenous *vps-41* transcripts and enhanced resistance to α-syn-induced neurodegeneration observed, in accordance with the established function of VPS-41.

Having demonstrated that α-syn-associated DA neurodegeneration displayed significant sensitivity to the presence or absence of SID-1, we set out to determine the consequences of attenuating the activity of SID-3 which, by extension of its known endocytic function, modulates levels of SID-1 in DA neurons and elsewhere in *C*. *elegans*. Although *sid-3* mutants have been shown to reduce the effectiveness of exogenous environmental RNAi in terms of systemic knockdown of corresponding genomic targets [23], it is unclear what effect reduction of SID-3 activity would have on neurodegeneration. The compound AIM-100 has been previously shown to inhibit the activity of ACK1 [31,32], the human ortholog of SID-3. ACK1 and SID-3 share domain structure, having similar tyrosine kinase domains, Src homology domains, and Cdc42/Rac interactive binding domains [23]. To determine if administration of AIM-100 could reduce the general effectiveness of environmental RNAi, GFP was knocked down via RNAi bacterial feeding. In these experiments, animals of a strain of *C*. *elegans*, HC46 [21], that overexpress nuclear-localized GFP in the large and readily observable body wall muscles, were reared on GFP-targeting RNAi bacteria, with or without AIM-100 (100 μM in 0.1% ethanol), and subsequently examined for GFP fluorescence. We predicted that AIM-100 would prevent GFP silencing via SID-3 inhibition. Indeed, animals that were fed control RNAi bacteria (empty vector) and not exposed to AIM-100 (ethanol solvent only) displayed robust expression of GFP in the body wall muscles (Fig 2A and 2D). When GFP was knocked down in animals not exposed to AIM-100, the GFP fluorescence was silenced in the body wall muscles to a large degree, as expected (Fig 2B and 2D). When GFP was targeted for knockdown in worms that were exposed to AIM-100, GFP was unable to be silenced in the body wall muscles to the same extent and exhibited significantly more GFP pixel intensity and noticeably brighter, more abundant fluorescence (Fig 2C and 2D). Therefore, a drug-induced reduction in SID-3 activity via AIM-100 diminishes the efficacy of exogenous RNAi knockdown, consistent with prior studies targeting GFP in the body wall muscles in genetic mutants of *sid-3* [23].

To determine if AIM-100 administration could reduce the effectiveness of exogenous RNAi in DA neurons overexpressing α-syn, we used an α-syn transgenic strain that enables the selective knockdown of genes only in the DA neurons. This strain (UA196) overexpresses human wildtype α-syn from a chromosomally integrated multicopy transgene in a *sid-1* mutant background, and concomitantly overexpresses *sid-1* from the DA neuron-specific *dat-1* promoter thereby restoring delimited RNAi sensitivity to just the DA neurons [33]. These worms were fed either empty vector (negative control) RNAi bacteria or RNAi bacteria producing dsRNA targeting *vps-41*, an established internal control which consistently enhances neurodegeneration due to a reduction in endolysosomal trafficking, an essential process for DA neuron health [34,35]. We carried out these RNAi assays with the addition of AIM-100, predicting that worms grown on *vps-41* RNAi bacteria in the presence of AIM-100 would display significantly less neurodegeneration compared to RNAi in the solvent-treated group. At day 4 post-hatching, knockdown of *vps-41* significantly enhanced neurodegeneration when compared to empty vector RNAi controls in the solvent control group (Fig 2E). However, knockdown of *vps-41* did not enhance neurodegeneration in the AIM-100-treated group (Fig 2E). Very similar results were obtained when the same experiment was performed on day 7 post-hatching (Fig 2F). In all, these combined results indicate that inhibition of SID-3 function by AIM-100 reduces the effectiveness of exogenous RNAi in both a non-neuronal tissue (body wall muscles) and in DA neurons (Fig 2G). Given that AIM-100 is established as both a potent and selective inhibitor of mammalian ACK1 [25,36], these data on the sole worm ACK1 ortholog, SID-3, indicate that this non-receptor tyrosine kinase represents a putative druggable target to modulate neurodegeneration through the endocytic regulation of small RNA uptake.

### Modulation of α-syn-induced DA neurodegeneration by *mir-2*

**Fig 3:**
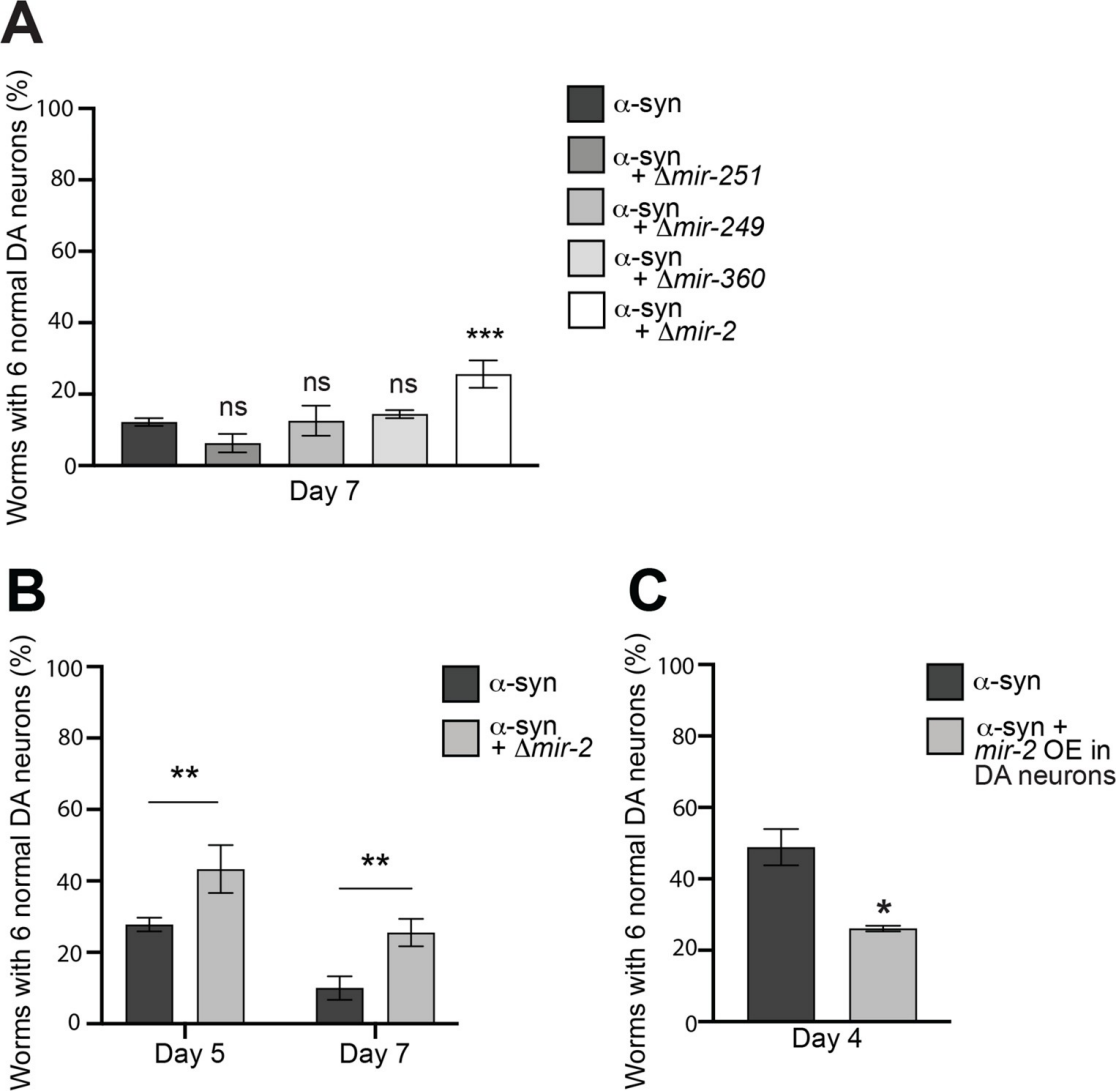
Modulation of α-syn-induced DA neurodegeneration by *mir-2*. **(A)** DA neurons were scored for degeneration on day 7 post-hatching. Values represent mean + S.D. (n = 30 worms per genotype per replicate, 3 independent replicates). One-Way ANOVA with Dunnett’s *post hoc* analysis was used to compare α-syn alone to α-syn crossed with Δ*mir-251(n4606)*, Δ*mir-249(n4983)*, Δ*mir-360(n4635)*, or Δ*mir-2 (gk259)*; ns P ≥ 0.05, *** P < 0.001. **(B)** DA neurons were scored for degeneration on days 5 and 7 post-hatching. Values represent mean + S.D. (n = 30 worms per genotype per replicate, 3 independent replicates). Two-Way ANOVA with Šídák’s *post hoc* analysis was used to compare α-syn alone to α-syn + Δ*mir-2(gk259)* at each time point; ** P < 0.01. (**C**) DA neurons were scored for degeneration on day 4 post-hatching. Values represent mean + S.D. (n = 30 worms per genotype per replicate, 3 independent replicates of 2 independent stable lines). Unpaired, two-tailed Student’s *t*-test was used to compare α-syn with *mir-2* overexpression (OE) in DA neurons (P_*dat-1*_::*mir-2*) to α-syn only controls; * P < 0.05.

Our previous findings revealed that a decrease in SID-3 function hindered RNAi when dsRNA was produced from an exogenous source (via bacterial feeding). Next, we wanted to determine if attenuating SID-3 activity could similarly impede gene silencing resulting from a naturally occurring miRNA in *C*. *elegans*. We were interested in identifying miRNAs that had validated targets with known cellular functions in critical regulatory mechanisms such as vesicle trafficking and Gα signaling that could mediate DA neuron survival. Importantly, four of these miRNAs had readily available knockout mutations that we crossed into α-syn worms. Of these, the *mir-2* mutant provided significant neuroprotection while the other miRNA knockout mutants (*mir-251*, *mir-249*, and *mir-360*), did not (Fig 3A). An analysis of miRNA evolution identified miR-2 as an epigenetic modifier that was likely to target neural genes, and multiple mature miR-2 sequences appear conserved between *C*. *elegans* and *D*. *melanogaster* [37]. The protection afforded by the *C*. *elegans mir-2* mutation was observed at both days 5 and 7 post-hatching in α-syn-expressing worms (Fig 3B). This suggests that an absence of epigenetic silencing of target gene expression by *mir-2* is beneficial for DA neurons in overcoming the stress from α-syn. It therefore follows that transgenic nematodes engineered to overexpress the *mir-2* pre-miRNA, exclusively in the DA neurons along with α-syn and GFP, display an increase in neurodegeneration, as was observed when compared to the α-syn background alone (Fig 3C).

### RNAi knockdown of conserved targets of *mir-2* enhances α-syn-induced DA neurodegeneration

**Fig 4:**
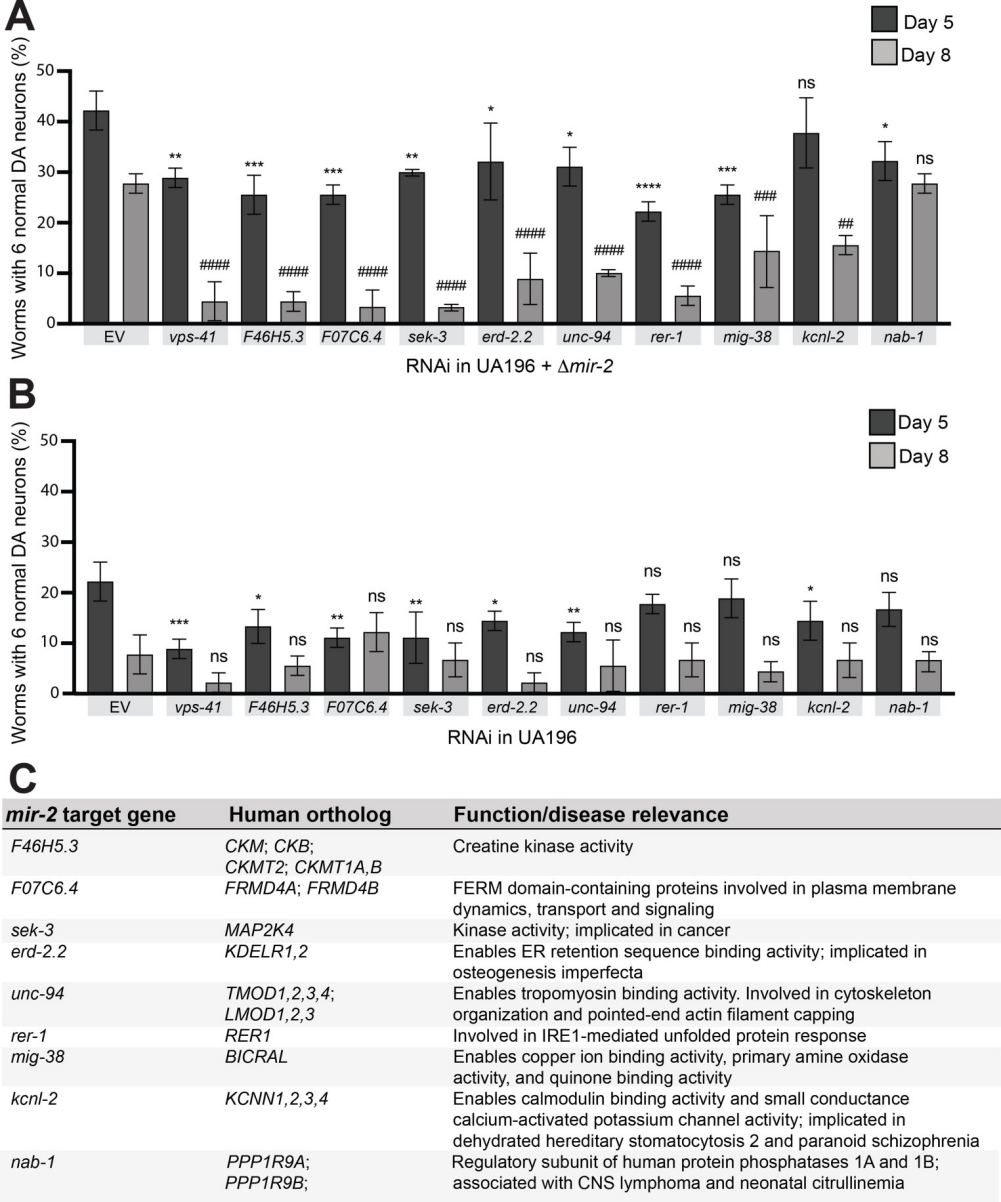
Modulating *mir-2*-dependent suppression of target gene expression levels alters α-syn-induced DA neurodegeneration. (**A, B**) DA neurons were scored for degeneration on days 5 and 8 post-hatching. RNAi was performed in a RNAi sensitive α-syn model with a *mir-2(gk259)* mutant background (**A**) and in the RNAi sensitive α-syn model that is *mir-2* wildtype (**B**). Nine validated targets of *mir-2* that have human orthologs were knocked down, along with a positive control, *vps-41*. Values represent mean + S.D. (n = 30 worms per genotype per replicate, 3 independent replicates). One-Way ANOVA with Dunnett’s *post hoc* analysis was used to compare RNAi knockdowns to empty vector (EV) controls at day 5 and 8 post-hatching. For day 5 analyses, asterisks (*) above bars indicate comparisons of each individual knockdown to EV; ns P ≥ 0.05, * P < 0.05, ** P < 0.01, *** P <0.001, **** P < 0.0001. For day 8 analyses, pound signs (#) above bars indicate comparisons to EV; ns P ≥ 0.05, ## P < 0.01, ### P <0.001, #### P < 0.0001. (**C**) A list of the nine targets knocked down via RNAi in (**A**) and (**B**), their human orthologs, and function.

To discern the role of *mir-2* effectors in neurodegeneration, individual target genes of *mir-2* were knocked down via RNAi. Only targets of *mir-2* that have human orthologs and have been previously validated as having a documented *mir-2-*regulated change in expression (by RNAseq and/or qPCR, as per Wormbase) were selected for knockdown. The 9 gene candidates examined code for proteins involved in a range of biological processes, including the unfolded protein response, cytoskeleton reorganization, and metal ion binding activity (Fig 4C). We reasoned that RNAi knockdown of these targets specifically in the DA neurons and in a *mir-2* mutant background, where their baseline of expression would not be repressed, would uncover individual contributors to the neuroprotection we observed in the absence of *mir-2* (Fig 4A). For this analysis, we employed the α-syn strain that allows for DA-neuron specific RNAi (UA196; used in Figs 1C, 2E and 2F) after crossing it to *mir-2* knockout mutants. The outcomes of this effort indicated that knockdown of 8 of the 9 validated *mir-2* targets resulted in an increased percentage of animals exhibiting DA neurodegeneration at day 5 post-hatching; a greater severity of degeneration was observed in these same targets by day 8 post-hatching (Fig 4A). These targets were also knocked down in UA196, which was wildtype for *mir-2* (Fig 4B). In this background, knockdown of 6 of the 9 validated *mir-2* targets resulted in an enhancement of neurodegeneration at day 5 post-hatching, whereas none of the *mir-2* targets displayed any significant effect on neurodegeneration at day 8 post-hatching following knockdown; the extent of neurodegeneration observed in the controls at this time point likely masked any significance (Fig 4B). These results are consistent with an interpretation that endogenous suppression of these genes by *mir-2* restricts their inherent neuroprotective capacity. Although these data reveal the cell autonomous effects of *mir-2* in DA neurons, we wanted to further explore the prospect that effectors of miRNA transport might also modulate neurodegeneration that could be attributed to cell non-autonomous effects for this specific miRNA.

### DA neurodegeneration by α-syn is enhanced by cell non-autonomous overexpression of *mir-2* and is modulated by AIM-100

**Fig 5:**
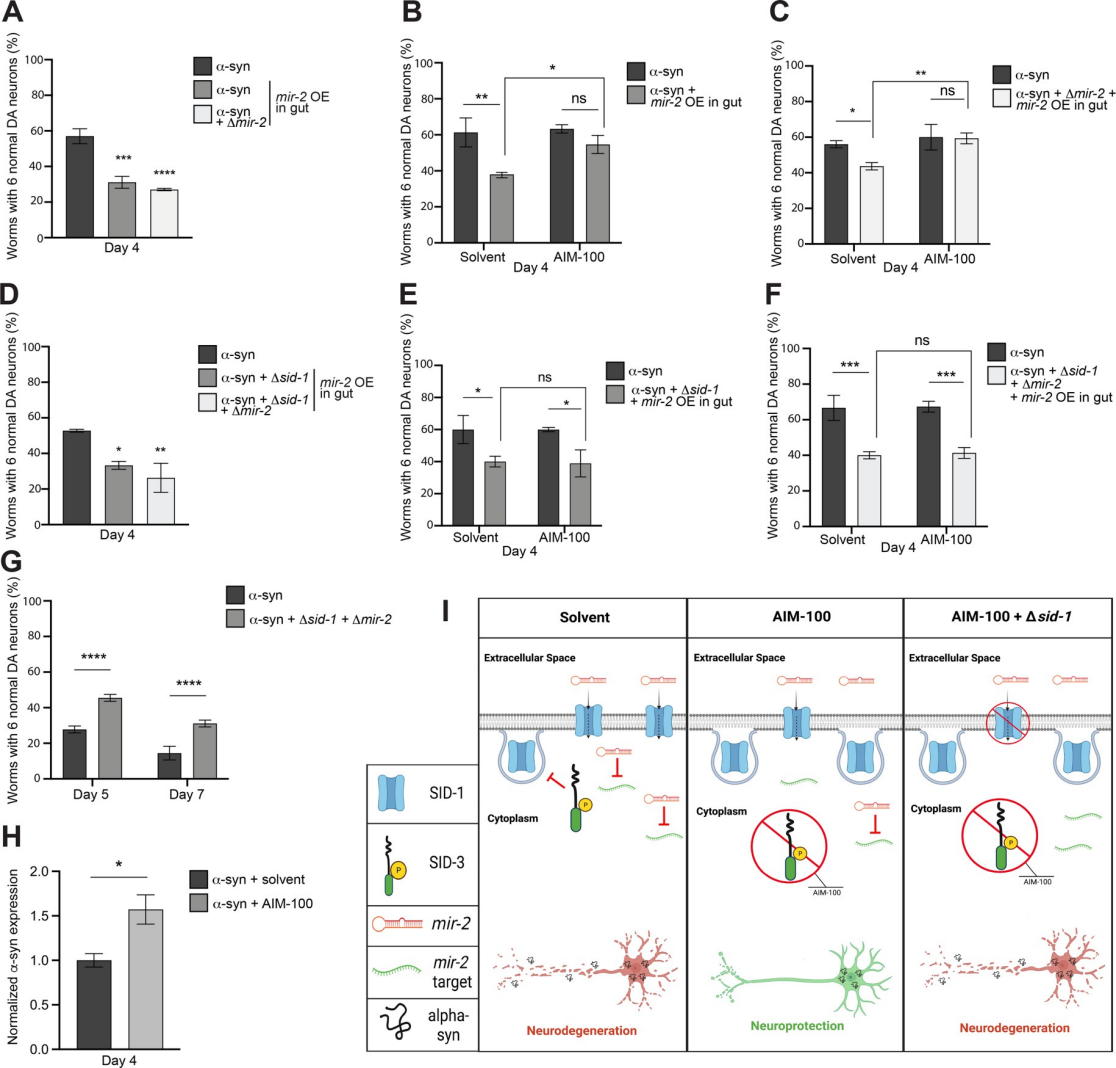
Intestinal overexpression of *mir-2* enhances DA neurodegeneration and is attenuated by AIM-100 when *sid-1* is wildtype. (**A, D**) DA neurons were scored for neurodegeneration at day 4 post-hatching in an α-syn model background with *mir-2* OE in the intestine (P_*ges-1*_::*mir-2*). In (**A**), *mir-2(gk259)* is examined (mutant and wildtype) while in (**D**), both *sid-1(pk3321)* and *mir-2(gk259)* mutants and wildtype are examined. Values represent mean + S.D. (n = 30 worms per genotype/group per replicate, 3 independent replicates of 3 independent stable lines). One-Way ANOVA with Dunnett’s *post hoc* analysis was used to compare worm classes where *mir-2* is OE in the intestine (P_*ges-1*_::*mir-2*) to the α-syn (with or without *sid-1* mutation) worm classes; * P < 0.05, ** P < 0.01, *** P <0.001, **** P < 0.0001. (**B, C, E, F**) DA neurons were scored for degeneration on day 4 post-hatching. Values represent mean + S.D. (n = 30 worms per genotype/group per replicate, 3 independent replicates of 1 independent stable line). Worms were either exposed to the 0.1% ethanol solvent control or AIM-100 (100 μM), and the α-syn control model or the α-syn model with *mir-2* OE in the intestine (P_*ges-1*_::*mir-2*) alone (**B**), with *mir-2(gk259)* (**C**), *sid-1(pk3321)* (**E**), or both *sid-1(pk3321)* and *mir-2(gk259)* mutants (**F**) were examined. Two-way ANOVA with Tukey’s *post hoc* analysis was used to compare all conditions to each other; ns P ≥ 0.05, * P < 0.05, ** P < 0.01, *** P <0.001. (**G**) DA neurons were scored for degeneration on days 5 and 7 post-hatching. Values represent mean + S.D. (N = 3; n = 30 worms, per genotype). Two-Way ANOVA with Šídák’s *post hoc* analysis was used to compare α-syn expressed in neurons to α-syn + *sid-1(pk3321)* + *mir-2(gk259)* at each time point; **** P < 0.0001. (**H**) Expression levels of α-syn in α-syn only worms and α-syn worms administered AIM-100. Expression is normalized to the α-syn only group. Total RNA was isolated at day 4 post-hatching. Unpaired, two-tailed Student’s *t*-test was used to compare α-syn + solvent to α-syn + AIM-100; * P < 0.05. (**I**) An illustrative triptych (created with Biorender.com) representing an interpretation of the results obtained for SID-3 regulation of *mir-2* import, modeling the differential impact of α-syn-induced DA neurodegeneration. The triptych displays conditions in which α-syn is expressed in DA neurons and *mir-2* is overexpressed (OE) in the worm intestine. In the left panel, *sid-1* and *sid-3* are wildtype, and SID-3 is depicted as inhibiting the endocytosis of SID-1, thus maintaining wildtype SID-1 levels for transport of *mir-2* and suppress the expression of its target genes, leaving DA neurons vulnerable to α-syn-induced neurodegeneration. The middle pane depicts these same animals treated with the potent and selective SID-3 inhibitor, AIM-100. When SID-3 is inhibited by AIM-100, evidence suggests that endocytosis of SID-1 is increased, resulting in a decrease of *mir-2* import into cells, and conversely increasing the abundance of upregulated transcripts that contribute to the DA neuroprotection observed. The right pane reflects the same scenario as does the middle, except under conditions where *sid-3* is wildtype, *sid-1* is mutant, and SID-3 is inhibited by AIM-100. If SID-3 activity is inhibited, increased internalization SID-1 from cell surfaces would be predicted, along with a corresponding decrease of *mir-2* import would be expected, aside from the fact that any SID-1 on cell surfaces is non-functional due to mutation. Strikingly, although *mir-2* import into cells via SID-1 is eliminated in this latter example, an enhancement of α-syn-induced neurodegeneration is still observed experimentally. This result suggests that an alternate mechanism of dsRNA uptake may exist and is revealed in the absence of functional SID-1.

Having determined that localized overexpression of *mir-2* in the DA neurons directly affected their survival in the presence of α-syn, we further explored the impact of *mir-2* produced from an indirect cellular source. We generated a strain which overexpressed *mir-2* (pre-miRNA) in the intestinal compartment (gut) under the control of the intestinal-specific promoter, *ges-1*, and crossed it into *C*. *elegans* expressing human, wildtype α-syn in the DA neurons. Unexpectedly, the tissue-specific overexpression of *mir-2* delimited to the worm gut led to a significant enhancement of DA neurodegeneration when compared to the α-syn background alone (Fig 5A). To ascertain whether intestinal *mir-2* overexpression was sufficient to induce the enhanced neurodegeneration observed, and that it was not dependent on the presence of an increase of endogenous *mir-2*, this strain was crossed into the *mir-2* knockout mutant previously examined. Interestingly, these animals retained an enhancement of neurodegeneration when compared to the α-syn background alone, akin to wildtype endogenous *mir-2* (Fig 5A). Therefore, the enhancement of DA neurodegeneration as a result of the overexpression of *mir-2* appears to overshadow the inherent neuroprotective effect of the *mir-2* mutation itself. These results showcase the potency of *in vivo* epigenetic regulation by microRNAs across cellular boundaries in an intact metazoan. Specifically, the overexpression of *mir-2* from within the gut of *C*. *elegans* exerts a robust, cell non-autonomous effect on the survival of DA neurons already sensitized to neurodegeneration by α-syn-induced neurotoxicity. In a theoretical variation on this scenario, an analogous effect might result from a microbiome-based source of dsRNA or inducer of host changes in small RNA expression that could, in turn, modulate susceptibility or resilience to neuron survival.

To determine if the previously observed neuroprotective effects of SID-3 inhibition could influence the enhanced neurodegeneration induced by intestinal *mir-2* overexpression, we administered AIM-100 to these animals. In the solvent control group, *mir-2* overexpression in the gut enhanced neurodegeneration in accordance with previous results (Fig 5B). In contrast, animals in the AIM-100-treated group no longer exhibited enhanced neurodegeneration as an outcome of *mir-2* overexpression (Fig 5B). A very similar outcome was observed when these same strains were crossed into the *mir-2* mutant background: overexpression of *mir-2* no longer enhanced neurodegeneration in AIM-100-treated animals (Fig 5C). The observation that intestinal *mir-2* overexpression increases α-syn-mediated neurodegeneration but is unable to do so when AIM-100 is administered, suggests that the effects of intestinal *mir-2* overexpression are at least partly dependent on SID-3 function in blocking endocytic recycling of plasma membrane proteins, such as SID-1. Likewise, the neuroprotection observed in response to SID-3 inhibition by AIM-100 might be attributable to a decrease in *mir-2* transport, thereby limiting silencing of its targets (Fig 5I).

To directly evaluate the effect of α-syn-induced DA neurodegeneration within the context of *sid-1* loss in worms overexpressing *mir-2* from within the intestine, these animals were crossed into the *sid-1* mutant background, and DA neurons were scored for neurodegeneration (Fig 5D). Surprisingly, when either *sid-1* mutants or *sid-1; mir-2* double mutants were crossed into *C*. *elegans* overexpressing *mir-2* from within the intestine (α-syn^*mir-2* OE gut^), the increased neurodegenerative effect previously observed remained significant (Fig 5D). This is especially noteworthy since *sid-1; mir-2* double mutants in the α-syn background are robustly neuroprotective compared to α-syn only controls, indicating that the neuroprotection conferred by these combined factors is insufficient to combat neurodegeneration resulting from overexpression of *mir-2* in the intestine (Fig 5G).

To determine if pharmacological inhibition of SID-3 impacted this observed cell non-autonomous effect on DA neurodegeneration in a manner independent of SID-1, we exposed either *sid-1* mutants or *sid-1; mir-2* double mutants in the α-syn^*mir-2* OE gut^ background to AIM-100. Both these sets of *sid-1*-deficient animals exhibited significantly enhanced neurodegeneration when compared to α-syn worms alone in the solvent controls (Fig 5E and 5F). When these same animals were exposed to AIM-100, intestinal overexpression of *mir-2* still led to significant enhancement of α-syn-induced DA neurodegeneration (Fig 5E and 5F). These results collectively suggest that the DA neurodegeneration observed reflects the import of *mir-2* into DA neurons and is a potential consequence of the subsequent silencing of *mir-2* target genes. Significantly, the increased neurodegeneration observed in response to *mir-2* overexpression in the intestine occurs in the absence of SID-1 transporter function and SID-3 inhibition by AIM-100 (Fig 5I).

We used RT-qPCR to determine if administration of AIM-100 altered α-syn levels and could potentially account for the reduction of enhanced DA neurodegeneration observed (Fig 5B). Interestingly, it was found that administration of AIM-100 to α-syn worms at day 4 post-hatching increased α-syn expression compared to solvent controls (Fig 5H). Importantly, AIM-100 is not neuroprotective from α-syn-induced neurodegeneration on its own at the time post-hatching that these experiments are conducted (day 4) (Fig 5B); a stage logistically necessitated to reveal any potential enhancement of neurodegeneration originating from *mir-2* overexpression, since α-syn worms are increasingly neurodegenerative with age. These results suggest that, despite an apparent increase in α-syn expression, the administration of AIM-100 protects DA neurons even with an additive effect on neurodegeneration imposed by *mir-2* overexpression. While an increase in α-syn transcripts in the presence of AIM-100 at this stage was not consequential for neurodegeneration, the mechanism underlying this observation warrants further investigation.

### Transcriptomic comparison of transgenic α-syn animals with and without *sid-1*

**Fig 6:**
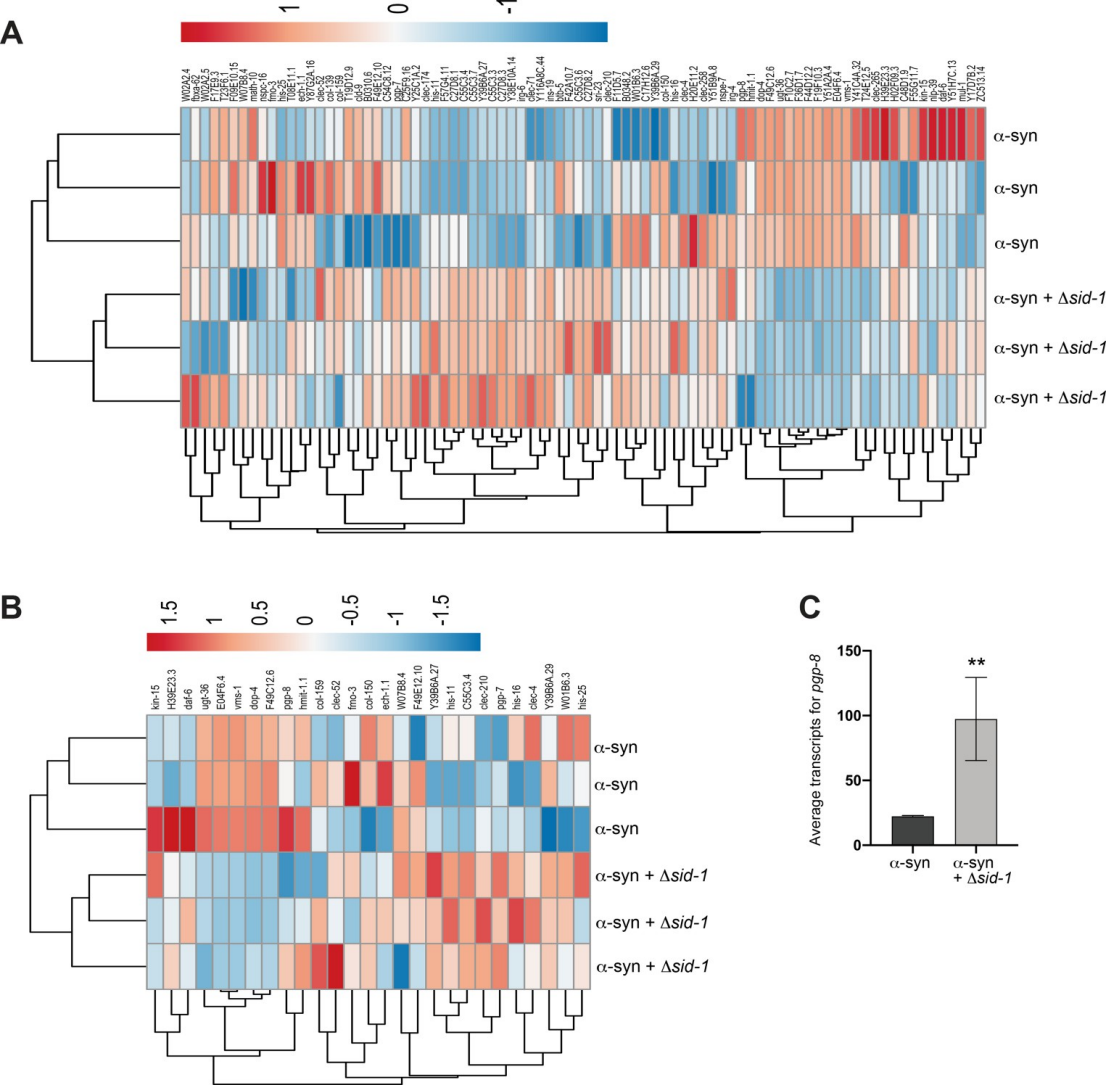
Transcriptomic analysis reveals DEGs between α-syn and α-syn + Δ*sid-1* worms. (**A, B**) Heatmaps depicting regulatory status of the DEGs (columns) from the transcriptomic analysis comparing α-syn and α-syn + Δ*sid-*1 groups (rows). Both the α-syn and α-syn + Δ*sid-1* groups constitute 3 replicates each. RNA was isolated at day 6 post-hatching. (**A**) Heatmap of all 84 DEGs associated with this analysis; (**B**) depicts the regulatory status of the 27 DEGs with human orthologs. (**C**) *pgp-8* mRNA transcript levels in the α-syn background alone and the α-syn background with the *sid-1* mutation, identified from the transcriptomic analysis between the α-syn and α-syn + Δ*sid-1* groups. Both groups constitute 3 replicates each. The DESeq2 program with the Benjamini-Hochberg method was used to obtain adjusted p-values (Novogene Corporation, Inc.); ** P(adjusted) < 0.01.

We have shown that *sid-1* and *sid-3* mutants promote the ability of DA neurons to combat α-syn-associated neurotoxicity. To determine which genes, pathways, or biological processes may be contributing to the favorable effect on DA neurons observed in *sid-1* mutants, a comparative transcriptomic analysis was performed on our transgenic α-syn (wildtype, multicopy, human) animals with and without the *sid-1* mutation. This analysis revealed a total of 84 Differentially Expressed Genes (DEGs), 74 of which were upregulated in α-syn worms with the *sid-1* mutation and 10 of which were downregulated, when compared to the α-syn background strain containing functional *sid-1* (Fig 6A). This bias towards DEGs being upregulated in animals with the *sid-1* mutation could be expected, since the expression of target genes normally suppressed by endogenous dsRNAs would no longer be silenced in the absence of the SID-1 transport function.

Out of the 84 DEGs identified, 27 had human orthologs, 22 of which were upregulated in *sid-1* mutants, and 5 of which were downregulated (Fig 6B). These DEGs encode products predicted to function in a myriad of biological processes, including transmembrane transport, metabolism, cell signaling, stress response pathways, transcriptional regulation, proteolysis, and neuronal function (Table 1). From the 27 DEGs with human orthologs, 18 (67%) were found to have a prior association to human diseases (Table 1). Notably, 9 of these have a previously identified association with PD in some way (33%); these include *clec-4*, *clec-52*, *dop-4*, *ugt-36*, *hmit-1*.*1*, *F49C12*.*6*, *F49E12*.*10*, *W07B8*.*4*, and *H39E23*.*3*. Interestingly, 4 other DEGs (*col-159*, *fmo-3*, *ech-1*.*1*, and *E04F6*.*4*) have a prior relationship to Alzheimer’s disease (AD) (Table 1). This candidate enrichment provides increased confidence that our strategy successfully informs us about mechanistic processes impacted by *sid-1*, and reveals specific, evolutionarily conserved gene products that contribute to neuroprotection.

**Table 1:** Differentially Expressed Genes (DEGs): α-syn vs. α-syn + Δ*sid-1*.

Gene	log2Fold Change	P value (adjusted)	Human Ortholog(s)	Function / Disease Relevance
**Downregulated in Δ*sid-1* + α-syn**				
*vms-1*	2.50	3.6E-177	*ANKZF1*	Linked to prevention of proteotoxic stress by freeing stalled ribosomes [38]
*E04F6*.*4*	2.20	2.63E-12	*PLD3-5*	detect foreign nucleic acids and participate in immune inflammation; PLD3 LOF tied to AD [39]; PLD4 tied to microglial activation [40]
*dop-4*	3.12	7.78E-11	*HTR1A;* *D3R*	dopamine receptor in *C*. *elegans*; biogenic amine dysfunction; generalized anxiety disorder; periodic fever; Parkinson’s disease, dyskinesia
*ugt-36*	3.38	5.42E-5	*UGT1A1*,*3–7*, *8*,*9*,*10;3A1*,*2* *UGT2A1*	metabolic enzymes; a UGT was associated with detoxification of α-synucleinopathy in PD [41]
*F49C12*.*6*	2.46	1.80E-5	*TMEM144*	transport of carbohydrates across membrane; linkage found to Lewy body dementia [42]
**Upregulated in Δ*sid-1* + α-syn**				
*Y39B6A*.*27*	3.20	1.04E-68	*MFSD11*	Uncharacterized neuronal solute carrier/transporter
*fmo-3*	2.29	1.49E-14	*FMO1-5*	Oxidoreductases linked to drug metabolism; linked to neuroprotection [43]
*clec-210*	3.50	9.00E-9	*REG1A*	carbohydrate binding; regulates cell migration; affects innate immunity and apoptosis
*Y39B6A*.*29*	3.12	9.66E-9	*MFSD11*	Uncharacterized neuronal solute carrier/transporter
*pgp-7*	3.13	6.71E-8	*ABCB1*,*4*,*5*,*11*	ATP-binding transmembrane transporter; inhibits xenobiotics entry via gut or across blood/brain barrier; linked to autoimmune disorders and cancers [44]
*his-11*	4.2	9.62E-8	*H2BC18*	Nucleosome component
*his-25*	2.46	2.67E-6	*H3C15*	Nucleosome component
*clec-4*	2.75	1.12E-5	*TLL1*. *CLEC3A*, *B*	Calcium-dependent carbohydrate binding; associated with innate immunity and cell adhesion; CLEC3B mouse knockouts have PD symptoms [45]
*ech-1*.*1*	2.03	7.64E-5	*HADHA*	Enzymes involved in catabolism of fatty acids; deficiency linked to mitochondrial dysfunction [46]; implicated in AD
*clec-52*	2.89	1.56E-4	*CD302;* *PLA2R1; MRC1*,*2*	Carbohydrate binding; associated with innate immunity, microglia phagocytosis, antigen processing; PLA2R1 induces senescence by promoting ROS accumulation [47]; MRC1 expression inversely related to PD in a mouse model through altered microglial activity [48]
*hmit-1*.*1*	2.30	5.04E-4	*SLC2A13*	Proton/myo-inositol symporter expressed in human brain; GWAS-identified mutation in SLC2A13 linked to PD [49]
*col-159*	2.74	0.0017	*COL6A5* *MARCO*	human macrophage receptor, implicated in amyloid-beta binding; linked to AD by amyloid-beta clearance and neuroinflammation mediated by glial cells [50]
*daf-6*	2.27	0.0021	*PTCHD1*,*3*,*4*	transcriptional regulator: null mutants exhibit dysfunctions in neuron mediated behavior; mutations in PTCHD1 linked to autism and other cognitive disorders due to disruptions to synaptic function [51]
*pgp-8*	2.07	0.0026	*ABCB1*,*4*,*5*,*11*	ATP-binding transmembrane transporter; inhibits xenobiotics entering body via gut or crossing blood/brain barrier; implicated in autoimmune diseases and cancers [44]
*col-150*	2.32	0.0043	*SFTPD*	Surfactant in human respiratory function/innate immunity; Implicated in inflammation [52]
*kin-15*	2.36	0.0067	*FLT3;* *KIT;* *PDGFRA*, *B; CSF1R*	Membrane receptor kinase: human orthologs exhibit very diverse functions; implicated in multiple cancers
*W01B6*.*3*	2.68	0.0081	*MFSD8*	Lysosomal transporter: mutations in MFSD8 cause a heritable neurodegenerative disorder characterized by accumulation of lysosomal storage materials [53]
*F49E12*.*10*	2.49	0.0097	*FAXDC2*	Oxidoreductase with predicted fatty acid modification; FAXDC2 tied to differentiation of platelet-generating marrow cells; blood RNA levels of FAXDC2 exhibit efficacy as a PD biomarker [54]
*W07B8*.*4*	2.21	0.0105	*CTSB*	Lysosomal protease; associated with promoting oxidative stress and microglial neuroinflammation [55]; decreased levels have been associated with exacerbating glucocerebrosidase dysfunction and overall PD risk [56]
*C55C3*.*4*	4.82	0.0112	*FER*, *FES*	non-membrane bound tyrosine kinase; mediates responses of innate immune pathways and growth factors in humans
*H39E23*.*3*	2.28	0.0125	*SON;* *NEFH;* *CLIC6*	microtubule binding in worm DA neurons; NEFH is an intermediate filament component of the neuronal cytoskeleton; CLIC6 is an intracellular chloride channel associated with regulation of D2-like receptors; NEFH was identified by whole exome sequencing of PD patient DNA [57]
*his-16*	3.79	0.0487	*H2AC21*	Nucleosome component

The transcriptomic comparison of α-syn worms vs. α-syn worms in the *sid-1* mutant background revealed two DEGs with human orthologs that code for transmembrane transporter proteins that were upregulated in the *sid-1* mutants compared to the α-syn background alone: *pgp-7* and *pgp-8*. These genes encode P-glycoprotein (P-gp) family members with human orthologs that are ATP-binding cassette (ABC) transporters. This class of transmembrane transporters traditionally function as exporters and are involved in the efflux of toxins, lipopeptides and lipophilic drugs in eukaryotes, including humans [58, 59]. However, exceptions have been reported, as the polarity of P-gp transport is neither exclusively unidirectional nor is it limited to efflux. Animals mutant for *sid-1* in the α-syn background exhibited significantly higher levels of *pgp-8* mRNA transcripts (>2-fold; p = 0.0026) compared to the α-syn background with wildtype *sid-1* (Fig 6C).

### Combined loss of both SID-1 and PGP-8 function reduces the enhancement of neurodegeneration resulting from *mir-2* overexpression

**Fig 7:**
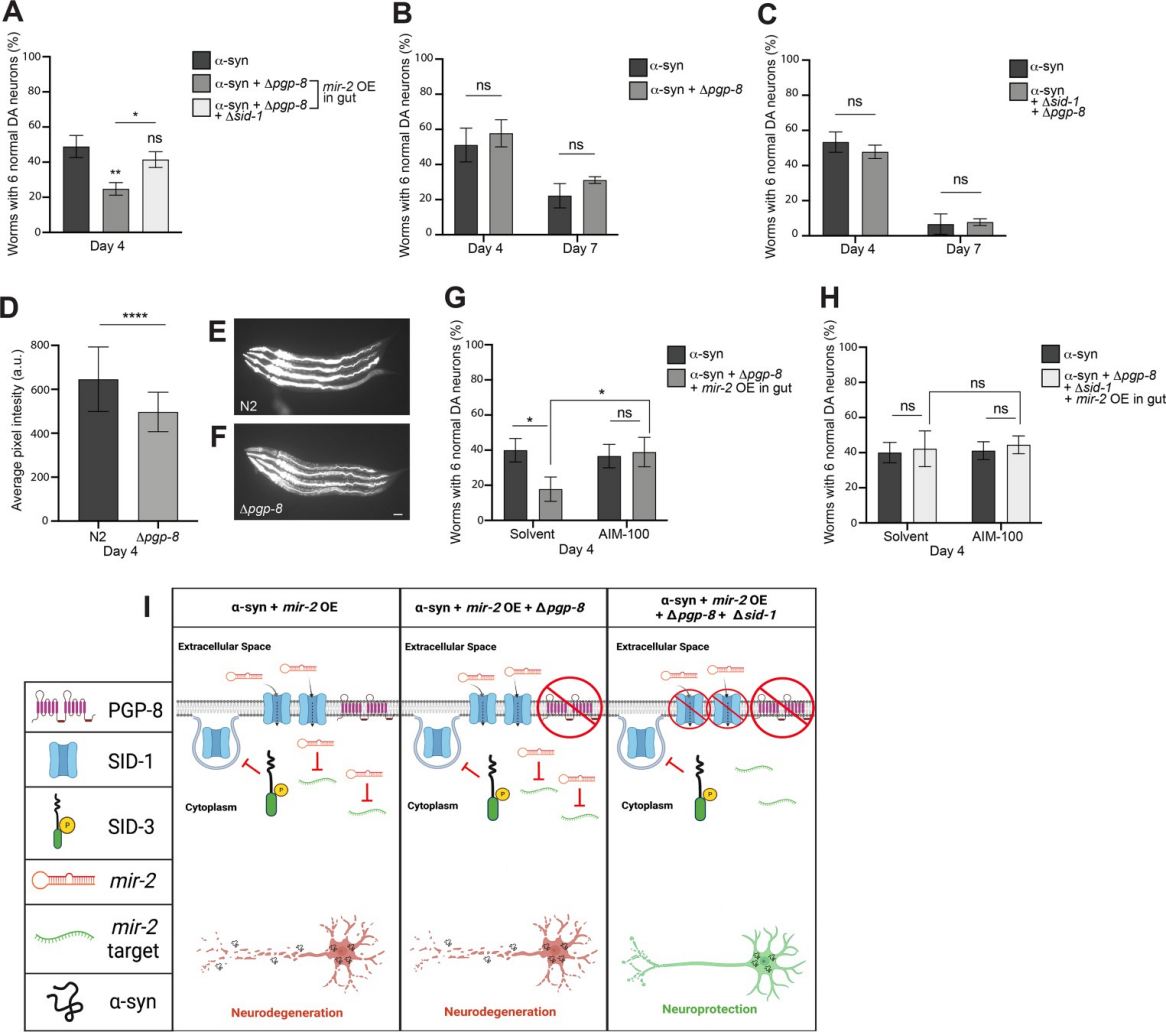
Loss of both SID-1 and PGP-8 functionality reduces *mir-2* OE-induced enhancement of neurodegeneration. (**A**) DA neurons were scored for neurodegeneration at day 4 post-hatching in an α-syn model background with *mir-2* OE in the intestine (P_*ges-1*_::*mir-2*), with a Δ*pgp-8(ok2489)* mutant and Δ*pgp-8(ok2489);* Δ*sid-1(pk3321)* double mutant. Values represent mean + S.D. (n = 30 worms per genotype/group per replicate, 3 replicates of 3 independent stable lines). One-Way ANOVA with Tukey’s *post hoc* analysis was used to compare all conditions to each other. Symbols above bars are in comparison to α-syn only controls while the straight line is a comparison of the bars indicated; ns P ≥ 0.05, * P < 0.05, ** P < 0.01. (**B**, **C**) α-syn control worms with *pgp-8(ok2489)* (**B**) or *pgp-8(ok2489); sid-1(pk3321)* (**C**) were analyzed for DA neurodegeneration at days 4 and 7 post-hatching. Values represent mean + S.D. (n = 30 worms per genotype per replicate, 3 independent replicates). Two-Way ANOVA with Šídák’s *post hoc* analysis was used to compare α-syn alone with the mutant displayed in the same graph; ns P ≥ 0.05. (**D**-**F**) N2 wildtype and *pgp-8(ok2489)* mutant worms following a 48-hour exposure to 10 μM of rhodamine 123. (**D**) Average pixel intensity was measured 48 hours post-hatching. Values represent mean + S.D. (n = 30 worms per genotype per replicate, 3 independent replicates). Unpaired, two-tailed Student’s *t*-test was used to compare N2 to *pgp-8(ok2489)*; **** P < 0.0001. Representative images of N2 (**E**) and *pgp-8* mutant (**F**) *C*. *elegans* (4 worms per panel, anterior to left) exhibiting fluorescence due to rhodamine 123 treatment. Scale bar, 50 μm. (**G, H**) DA neurodegeneration at day 4 post-hatching. Values represent mean + S.D. (n = 30 worms per genotype/group per replicate, 3 independent replicates of 1 independent stable line). α-syn control worms or the α-syn with *mir-2* OE in the intestine (P_*ges-1*_::*mir-2*) with *pgp-8(ok2489)* (**G**) or *pgp-8(ok2489); sid-1(pk3321)* (**H**) were either exposed to the 0.1% ethanol solvent control or AIM-100 (100 μM). Two-way ANOVA with Tukey’s *post hoc* analysis was used to compare all conditions to each other; ns P ≥ 0.05, * P < 0.05. (**I**) An triptych illustration (created with Biorender.com) reflecting an interpretation of the experimentally observed impact on α-syn-induced DA neurodegeneration caused by intestinal overexpression of *mir-*2 in either a *pgp-8* knockout mutant or a *pgp-8*; *sid-1* double mutant. In the left pane, SID-3 blocks the endocytosis of SID-1 on cell membranes, maintaining ample SID-1 on cell surfaces to transport *mir-2* into cells, allowing for *mir-2* target gene silencing and leading to enhanced neurodegeneration. The middle pane displays the situation when α-syn is expressed in DA neurons and *mir-2* is OE in the intestine, while *sid-1* is wildtype and *pgp-8* is mutant. In this model, SID-3 blocks the endocytosis of SID-1 on the plasma membrane, resulting in the transport of *mir-2* for target gene silencing and the enhanced neurodegeneration observed experimentally. It is presumed that a loss of PGP-8 function does not influence *mir-2* import into cells, since SID-1 remains functional. The right pane is a model for when both *sid-1* and *pgp-8* are mutant in animals expressing α-syn in the DA neurons, with *mir-2* being overexpressed in the intestine. Here we hypothesize that *mir-2* might not be imported into cells either due to a mutation that renders SID-1 non-functional, or by the enigmatic process by which PGP-8 may influence *mir-2* import into cells, also associated with a mutation rendering it non-functional. In either case, less or no *mir-2* import into cells results, and instead, leads to an infusion of target gene transcription that contributes to the observed neuroprotection.

Given the availability of a *pgp-8* deletion mutant, we reasoned it worthwhile to investigate the prospect that the upregulation of *pgp-8* in response to *sid-1* mutation potentially represented a form of compensatory response to systemic SID-1 deficiency. Therefore, α-syn^*mir-2* OE gut^ worms were crossed to animals mutant for *pgp-8*, as well as to *pgp-8*; *sid-1* double mutants and were subsequently scored for changes in α-syn-induced DA neurodegeneration. When *pgp-8* alone was mutant, intestinal *mir-2* overexpression significantly enhanced neurodegeneration compared to the α-syn only background (Fig 7A). However, when *pgp-8* and *sid-1* were both mutated in this same background, the degenerative effect of intestinal *mir-2* overexpression was negated (Fig 7A). In the absence of *mir-2* overexpression, neither *pgp-8* mutants nor *pgp-8*; *sid-1* double mutants exhibited any significant changes in α-syn-induced DA neurodegeneration (Fig 7B and 7C). These results suggest that the absence of both SID-1 and PGP-8 function disrupts cellular entry of intestinal *mir-2* and consequentially impedes silencing of the neuroprotective targets of *mir-2*, as opposed to what was observed when either *sid-1* or *pgp-8* was independently mutant in α-syn^*mir-2* OE gut^ worms (Fig 7I).

Having identified a requirement for PGP-8 function for α-syn^*mir-2* OE gut^ DA neurodegeneration in the absence of functional SID-1, we wanted to explore the prospect that PGP-8 may be functioning as an importer, as opposed to a more common role for P-gps in efflux of a broad range of substrates from within cells. To examine this, we exposed N2 wildtype animals and worms with the *pgp-8* mutation to rhodamine 123, a fluorescent dye that localizes to mitochondria and is a known conserved substrate for P-gp ABC transporters [60]. According to the *C*. *elegans* Neuronal Gene Expression Map and Network (CeNGEN) [61], PGP-8 is expressed in body wall muscle cells and many neurons in *C*. *elegans*, including the DA neurons. Therefore, we hypothesized that PGP-8 may exhibit influx activity, and therefore *pgp-8* mutants might display an overall decrease in rhodamine fluorescence. The quantified average pixel intensity of rhodamine 123-induced fluorescence displayed by *pgp-8* mutant worms was significantly decreased compared to that of N2 worms exposed to this dye (Fig 7D–7F). When taken in the context of the results of the functional genetic analyses performed, these observational data, albeit limited in scope and not specific to DA neurons, suggest the potential exists for PGP-8 to function in the influx of substrates.

To explore this further, we conducted a similar set of experiments using AIM-100 to inhibit SID-3 in worms with the α-syn^*mir-2* OE gut^ background that also had either the *pgp-8* mutation alone or both *pgp-8* and *sid-1* mutated. When only *pgp-8* was mutant (solvent group), *mir-2*-induced enhancement of α-syn-mediated DA neurodegeneration was observed (Fig 7G). In contrast, when AIM-100 was administered to these same worms, α-syn-mediated neurodegeneration was attenuated (Fig 7G). This implies that loss of PGP-8 function was of little consequence in the presence of functional SID-1, as AIM-100 treatment exerts the same neuroprotective effect on *mir-2*-induced neurodegeneration as was previously observed (Fig 5B and 5C). When both *pgp-8* and *sid-1* were mutant in the α-syn^*mir-2* OE gut^ background (solvent group), the DA neurodegeneration observed was consistent with the effect seen with untreated α-syn animals containing both *pgp-8* and *sid-1* mutations; no significant alteration in neurodegeneration was seen (Fig 7H). Administration of AIM-100 to *pgp-8*; *sid-1* double mutants, intestinal *mir-2* overexpression had no additive effect on α-syn-induced DA neurodegeneration (Fig 7H). This indicates that the inhibition of SID-3 activity by AIM-100 attenuates the cell non-autonomous neurodegenerative effect of *mir-2* overexpression, but only in the presence of functional SID-1, and inhibition of SID-3 is independent of *pgp-8* status. The observed dependence on *pgp-8* for *mir-2* to enhance DA neurodegeneration when overexpressed from the intestine suggests an alternative pathway, independent of *sid-1*, can plausibly regulate epigenetic modulation of DA neurodegeneration. Transcriptional changes in response to *sid-1* depletion clearly exert functional effects in how dsRNAs like *mir-2* (and other miRNAs) convey epigenetic signals to silence genes (Fig 7I). Further investigations of the other genes found to be differentially expressed in *sid-1* mutants are poised to reveal more mechanistic insights.

## Discussion

Whereas precise causes of PD are complex and unresolved, this movement disorder is unequivocally a combined consequence of genetic predisposition and epigenetic response to exogenous (i.e., toxins) and/or intrinsic environmental (i.e., microbiome) factors. It is to be expected that an increased susceptibility to neurodegenerative states accompanying a chronic source of cellular stress, such as expression of a chromosomally integrated multicopy α-syn transgene, further exacerbated with time and aging, evokes inherent organismal responses. Indeed, numerous studies across a wide range of cellular and animal systems have examined the transcriptional response to overexpression of α-syn or other genes, mutations in those loci, various PD-associated toxins, addition of pre-formed α-syn fibrils, and more. None of these models enable rigorous experimentation aimed at understanding the consequences on neurodegeneration that may arise from disruption and changes in the efficacy of small RNA transport and the systemic effects of gene silencing, as has proven possible using *C*. *elegans* [21]. Recent results of transcriptional profiling experiments using a different *C*. *elegans* α-syn model documented extensive changes in the expression of miRNAs and piRNAs [16]. Our present study diverges from further descriptive cataloging of α-syn-induced gene expression to instead consider a different, more mechanistic question. Specifically, how is it that small RNAs exert a functional impact as modifiers of the degeneration of DA neurons?

Here we have demonstrated that the DA neurons of *C*. *elegans* are receptive and responsive to epigenetic regulation by endogenous, exogenous, cell autonomous, and cell non-autonomous sources of dsRNA. In discerning that established components of the systemic gene silencing machinery of *C*. *elegans*, SID-1 and SID-3, can function as effectors of α-syn-induced DA neurodegeneration, we provide a roadmap for future investigation of conserved targets of their activity in modulating neurodegenerative disease, including numerous previously unrecognized neuroprotective gene products.

It is important to note that even in *sid-1* mutants, lacking systemic dsRNA transporter function, the process of endogenous gene silencing appears to remain intact within cells that themselves transcribe dsRNAs. Thus, the abolishment or inhibition of gene silencing in *sid-1* or *sid-3* mutants only applies to genes that are silenced by mobile dsRNAs that originate outside of target cells and require transport into the cytoplasm. Moreover, it appears as though the neuroprotective benefit DA neurons receive from reduced gene silencing in α-syn worms is largely dependent on SID-1 transporter function. A caveat to our studies is that we only examined a single allele, each, of *sid-1* and *sid-3*. In the future, we envision extending our work to include additional alleles of these genes to strengthen our conclusions [13].

Contrary to the prevailing dogma in the *C*. *elegans* field [62], the levels of SID-1 present in the DA neurons are clearly not too low to exert a functional effect that can be revealed either by *sid-1* knockout and/or dsRNA expression from within the animal. While neuronal *sid-1* expression levels are unequivocally lower on average when compared to other cell types, recent data that have emerged from CeNGEN shows that *sid-1* is expressed in DA neurons [61]. Perhaps the comparatively limited levels of basal *sid-1* expression are reflected by the observation that, in *sid-1* mutant animals, the only significant reduction in neurodegeneration was more limited to later in life, when the progressive effect of α-syn-induced DA neurodegeneration is typically most extensive. However, *sid-1* mutants still exhibit significantly improved behavioral functionality at earlier timepoints, and as demonstrated by the restoration of DA neurotransmission via the BSR.

The protection from α-syn neurotoxicity observed in *sid-3* mutants was more robust at all ages examined. SID-1 is still present in these animals, but it is subjected to increased endocytic recycling at the plasma membrane in the absence of SID-3 non-receptor tyrosine kinase activity. The difference in neuroprotection observed in *sid-3* mutants in comparison to *sid-1*; *sid-3* double mutants, specifically at timepoints when *sid-1* mutants alone showed little or no neuroprotection, is not unexpected. This is likely a reflection of SID-3 also having an important SID-1- and dsRNA-independent function as a regulator of the basal endocytosis of the *C*. *elegans* DA transporter, DAT-1. As was previously described, ACK1 (the human ortholog of SID-3) regulates internalization of DAT on the plasma membrane of DA neurons to modulate synaptic dopamine levels and neurotransmission [25]. Accordingly, others and we have also reported that *dat-1* mutants in *C*. *elegans* protect DA neurons from degeneration [63,64]. Whereas *dat-1* gene expression is limited to the DA neurons, the *sid-3* gene is more broadly expressed, as is *sid-1*. Thus, it is tempting to speculate that this duality of transporter regulation observed within the DA neurons is a unique evolutionary confluence of function inherent to SID-3. DA biosynthesis, packaging, synaptic release and reuptake, as well as its reception, metabolism, and function are collectively well established as a tightly regulated integration of evolutionarily conserved processes. Therefore, the endocytic control of both neurotransmitter and dsRNA transporter function by SID-3 represents a nexus of genetic and epigenetic convergence that promotes a capacity for dynamic dopaminergic responsivity to both internal and external influences on an organism.

It is interesting to consider other partners of SID-3 endocytic regulation, especially given the range of substrates ACK1 is known to phosphorylate in mammalian cells, where it is implicated in tumor survival in a variety of cancers [65]. Clearly, the functional impact of SID-3 is not limited to its role with systemic RNAi and SID-1. In this regard, research into mechanisms of viral infection of *C*. *elegans* have proven insightful in expanding our understanding of SID-3 function. Specifically, genetic screens conducted to uncover effectors of Orsay virus infection identified the *nck-1* and *wsp-1* genes, which encode the worm orthologs of known interactors of mammalian TNK2/ACK1 necessary for infection by multiple forms of picornaviruses [66,67]. Interestingly, *sid-3* mutants display a ~100-fold decrease in viral RNA load and exhibit a loss of repression of antiviral response genes [26]. While overexpression of a pre-miRNA from *within* the intestine, as we have done with *mir-2*, does not necessarily equate to a virus entering the animal, the mechanistic parallels are intriguing, as non-enveloped “naked” RNA viruses also utilize endosomal pathways for infection. Since RNAi evolved as a natural defense from viral infection, continued investigations are likely to converge with studies involving dsRNA translocation and miRNA-associated gene silencing in *C*. *elegans*.

The comparative transcriptomic analysis performed using transgenic α-syn animals, either with or without the *sid-1* mutation, provides a window into putative candidate gene products that exert a differential function when SID-1 dsRNA transporter activity is absent. In this regard, among the list of 27 DEGs with human orthologs identified, the increased expression of the *pgp-7* and *pgp-8* genes were of particular interest. Both these genes encode highly conserved members of the P-glycoprotein (P-gp) ABC transporter family, primarily known for their broad substrate specificity in the translocation of a variety of molecules across the plasma membrane of cells. P-gp transporters are generally classified as facilitating the efflux of toxins, drugs, lipophilic peptides, and other small molecules out of cells. Nevertheless, examples of ABC transporters functioning to import substrates exist and are present in both plants and animals, including humans [68,69]. Both *pgp-7* and *pgp-8* are orthologs of human *ABCB1*. Indeed, even this archetypical P-gp, the ABCB1/MDR1 multidrug resistance efflux transporter, has been shown to function in the influx of substrates from the epidermis to the skin of mice, whereas *mdr1 (-/-)* mice lack the deposition of epidermally derived molecules [60]. In a feat of protein engineering, it was recently demonstrated that the directionality of transport could be reversed from efflux to influx by mutagenesis of conserved residues in MDR1/ABCB1 [70]. Knowing that *pgp-8* is an ortholog of *ABCB1*, bolsters our nascent hypothesis that PGP-8 could function as an importer. Precedent exists for the reversal of astrocytic solute transporter direction in response to dopamine, triggering the release, instead of uptake, of glycine [71]. While speculative, one can envision a scenario whereby PGP-8 could potentially export a lipophilic small molecule or peptide that induces neighboring cells (i.e., CEPsh, glial cells that ensheath the anterior-most DA neurons) to respond to the export of such a factor that could, in turn, impact neuronal dsRNA import. Going forward, *in vitro* biochemical assays to examine directionality of PGP-8 and/or functional interactions with other transporters represent worthwhile directions.

Among the earlier mechanistic observations on RNAi in *C*. *elegans* was a report that demonstrated that the efficacy of dsRNA gene silencing showed a requirement for members of the P-gp ABC transporter family to be expressed in the same cells as the gene being targeted for knockdown [72]. Through a screen of 43 different ABC transporters for which mutants had been isolated, it was determined that a total of 9 (about 30%) of these genes non-redundantly influenced RNAi activity. The *C*. *elegans* genome encodes over 60 members of the ABC transporter family, most of which have not been extensively characterized with respect to function, including PGP-8, which was not among the genes tested at the time of this prior study, but it has been reported to exhibit an enriched expression in neurons [61]. Interestingly, over 100 genes have been shown to play a role in the network of genes comprising RNAi function in *C*. *elegans*, yet the SID proteins and these ABC transporters are among very few of those that localize to membranes [73]. To the best of our knowledge, no precedent exists for the translocation of small RNAs by ABC transporter proteins, and we are reluctant to speculate that our observations are a result of such a mechanism. Whatever substrate(s) of PGP-8 are involved with its effect on either the systemic spread of RNAi, or in a more localized role in DA neurons, this relationship merits further attention.

Still, if specific gene products functionally compensate for SID-1 in its absence, then dsRNA import into cells would be lost, or at least diminished when they too were mutant. It was therefore notable that the combined genetic depletion of both *sid-1* and *pgp-8* in double-mutant animals abolished the ability of an intestinally expressed miRNA, *mir-2*, to significantly enhance neurodegeneration. Whereas when either *sid-1* or *pgp-8* were individually mutant, the cell non-autonomous overexpression of *mir-2* had no influence on α-syn-induced DA neurodegeneration. Arguably, the most fascinating result from this study was that, even in a *sid-1* mutant background, intestinal expression of *mir-2* was still able to enhance the degeneration caused by α-syn in DA neurons. Taken on its own, this could suggest that *mir-2* is silencing target genes locally, in the intestinal cells, which would then indirectly affect DA neuron degeneration. This would be an appealing explanation, as there is a growing precedent for gut-to-neuron signaling as being a contributing factor in the etiology of PD [74,75]. In our case, this possibility is contested since AIM-100 administration, which decreases dsRNA import in all cells expressing *sid-3*, abolishes the ability of *mir-2* to enhance α-syn-mediated neurodegeneration. Furthermore, as it is known that SID-1 can import dsRNAs of at least up to 500 basepairs, overexpression of *mir-2* (pre-miRNA, 98bp) should be readily transported by SID-1 [76]. Taking this into account, this result remains enigmatic, since the ability of *mir-2* to access cells, silence target genes and subsequently affect α-syn-mediated neurodegeneration should be completely dependent on the presence of functional SID-1. Considering that knockout of both *pgp-8* and *sid-1* (*pgp-8*; *sid-1* double mutants) eliminates the enhanced neurodegeneration caused by intestinal *mir-2* overexpression, this may provide a clue to the organismal dynamics that are at play when dsRNA transport activity is compromised or depleted by mutation.

Since the transmission of *mir-2* appears to induce an increased susceptibility to neurodegeneration, this counterintuitively suggests that the basal expression levels of certain genes (i.e., targets of miRNAs) are maintained in a state that hinders the capacity of DA neurons to protect themselves. However, that premise represents an oversimplification of the integrated dynamics of the various cellular subcomponents involved in maintaining neuronal homeostasis. First, the enzymatic pathways involved in the biosynthesis of DA, as well as the cellular machinery responsible for vesicular packaging, trafficking, metabolism, synaptic release and reuptake are all tightly regulated processes conserved between worms and humans. Second, while the misfolding and oligomerization of α-syn, along with its concomitant impact on cellular dynamics are central to the underlying pathology of PD [77], the fact that the *C*. *elegans* genome does not naturally contain an α-syn homolog complicates the interpretation of why a loss or decrease of dsRNA-mediated gene silencing is beneficial in this context. Third, the levels of dopamine itself play a vital role in the selective vulnerability of DA neurons to a variety of potential sources of damage (i.e., oxidative, mitochondrial, lysosomal). In the case of PD, dosage-dependent misfolding and oligomerization associated with α-syn neurotoxicity introduces a significant impediment to neuronal homeostasis. We previously demonstrated this imbalance is further compounded *in vivo*, where evidence from both *C*. *elegans* and mouse α-syn models demonstrated a direct role for the dysregulation of DA levels in neurodegeneration because of the interaction of this neurotransmitter with α-syn [78]. Therefore, optimal DA neuronal functionality is dependent on a suite of factors that, if perturbed by any of a series of environmental, genetic, or epigenetic factors, can profoundly impact susceptibility to neurodegeneration. In considering the pivotal roles that both SID-1 and SID-3 have in the orchestration of epigenetic regulation by small RNAs, systemically in *C*. *elegans*, it is not difficult to imagine the impact on DA neuron homeostasis and survival that we have observed.

This study offers a new perspective that positions the activity of SID-1 and SID-3, and their implicit regulation of epigenetic gene silencing, in a pivotal relationship with dopaminergic neurotransmission and neurodegeneration, specifically as it pertains to synucleinopathies like PD. Added support for this relationship has recently come from a pathology study showing that the human SIDT2 protein was found co-localized with a-synuclein in Lewy Bodies of patients with PD and Lewy Body Dementia [79]. While it is likely to vary substantially, the conceptual model that continues to emerge in this research may prove applicable to other cells and neuron subtypes in *C*. *elegans*. Furthermore, although stark anatomical differences and other complexities obviously preclude direct comparisons between organismal mechanisms of small RNA transport among highly diverse species, the existence of human orthologs of both SID-1 and SID-3 points to mechanistic commonalities that are yet to be discerned [31,32,80]. Likewise, even though the conservation of miRNA sequences and structures is limited between humans and worms, this does not nullify the value in being able to identify evolutionarily conserved genes that are targets of epigenetic regulation, and whose expression is influenced by the dsRNA transport machinery. Moreover, our results indicate that this approach represents a strategy to reveal conserved functional modifiers representative of potential therapeutic targets, previously unrecognized for their neuroprotective capacities. Further investigation toward fine tuning their expression as individual targets or, perhaps, as co-regulated factors with a combined potency, should be explored with the requisite sense of urgency to implement innovations of therapeutic benefit for neurodegenerative diseases.

## Materials and methods

### *C*. *elegans* strains

Experimental nematodes were reared and maintained on OP50-1 *E*. *coli* bacteria (unless otherwise noted) at 20°C under standard laboratory conditions [81]. A complete list of the strains used in this study are shown in S1 Table. The following strains were obtained from the CGC: N2, CB1112 (*cat-2*(*e1112*)), and RB1916 (*pgp-8(ok2489)*). Strain HC46 [*ccls4251*(P_*myo-3*_::GFP-NLS (nuclear localized), P_*myo-3*_::GFP-MITO (mitochondrial localization); *mls11*(P_*myo-2*_::GFP)] was a gift from Craig Hunter (Harvard). Integrated transgenic strain BY250 (*vtIs7* [P_*dat-1*_::GFP]) was a gift from Randy Blakely (Florida Atlantic Univ.). Integrated transgenic lines crossed into BY250 include: UA423 (*vtIs7*[P_*dat-1*_::GFP]; *sid-1*(*pk3321*). There were 3 separate integrated transgenic α-syn neurodegeneration models that were used in this study: UA44 (*baIn11*[P_*dat-1*_:: α-syn (human, wildtype), P_*dat-1*_::GFP]), UA372 (*baIn54*[P_*dat-1*_::α-syn (human, A53T mutation), P_*unc-54*_::tdTomato]), and UA196 (*baIn11*[P_*dat-1*_:: α-syn (human, wildtype), P_*dat-1*_::GFP]; *baIn33* [P_*dat-1*_::*sid-1*, P_*myo-2*_::mCherry]; *sid-1*(*pk3321*)).

Integrated lines and mutations crossed into UA44 include: UA415 (*baIn11*[P_*dat-1*_::α-syn (human, wildtype), P_*dat-1*_::GFP]; *sid-1*(*pk3321*)), UA416 (*baIn11*[P_*dat-1*_::α-syn (human, wildtype), P_*dat-1*_::GFP]; *sid-3*(*ok973*)), UA414 (*baIn11*[P_*dat-1*_::α-syn (human, wildtype), P_*dat-1*_::GFP]; *sid-1*(*pk3321*); *sid-3*(*ok973*)), UA418 (*baIn11*[P_*dat-1*_::α-syn (human, wildtype), P_*dat-1*_::GFP]; *mir-2*(*gk259*)), UA417 (*baIn11*[P_*dat-1*_::α-syn (human, wildtype), P_*dat-1*_::GFP];*sid-1*(*pk3321*); *mir-2*(*gk259*)), UA419 (*baIn11*[P_*dat-1*_::α-syn (human, wildtype), P_*dat-1*_::GFP]; *pgp-8*(*ok2489*)), UA436 (*baIn11*[P_*dat-1*_::α-syn (human, wildtype), P_*dat-1*_::GFP]; *sid-1*(*pk3321*); *pgp-8*(*ok2489*), UA444 *baIn11*[P_*dat-1*_:: α-syn, P_*dat-1*_::GFP]; *mir-251*(*n4606*), UA445 *baIn11*[P_*dat-1*_:: α-syn, P_*dat-1*_::GFP]; *mir-249*(*n4983*), UA446 *baIn11*[P_*dat-1*_:: α-syn, P_*dat-1*_::GFP]; *mir-360*(*n4635*). Integrated transgenic lines crossed into UA372 include: UA420 (*baIn54*[P_*dat-1*_:: α-syn (human, A53T), P_*unc-54*_::tdTomato]; *sid-1*(*pk3321*)), and UA421 (*baIn54*[P_*dat-1*_:: α-syn (human, A53T), P_*unc-54*_::tdTomato]; *sid-3*(*ok973*)). Integrated transgenic lines crossed into UA196 include: UA437 (*baIn11*[[P_*dat-1*_:: α-syn (human, wildtype), P_*dat-1*_::GFP]; *baIn33*[P_*dat-1*_::*sid-1*, P_*myo-2*_::mCherry]; *sid-1*(*pk3321*); *mir-2*(*gk259*)). Stable transgenic lines crossed into UA44 include: UA273 (*baIn11*[P_*dat-1*_::α-syn (human, wildtype), P_*dat-1*_::GFP]; *baEx161*[P_*dat-1*_::*mir-2* (pre-miRNA), P_*unc-54*_::mCherry)]), UA408 (*baIn11*[P_*dat-1*_::α-syn (human, wildtype), P_*dat-1*_::GFP]; *baEx226*[P_*ges-1*_::*mir-2* (pre-miRNA), P_*unc-54*_::tdTomato]), UA409 (*baIn11*[P_*dat-1*_::α-syn (human, wildtype), P_*dat-1*_::GFP]; *baEx226*[P_*ges-1*_::*mir-2* (pre-miRNA), P_*unc-54*_::tdTomato]; *mir-2*(*gk259*)), UA410 (*baIn11*[P_*dat-1*_::α-syn (human, wildtype), P_*dat-1*_::GFP]; *baEx226*[P_*ges-1*_::*mir-2* (pre-miRNA), P_*unc-54*_::tdTomato]; *sid-1*(pk3321)), UA411 (*baIn11*[P_*dat-1*_::α-syn (human, wildtype), P_*dat-1*_::GFP]; *baEx226*[P_*ges-1*_::*mir-2* (pre-miRNA), P_*unc-54*_::tdTomato]; *mir-2*(*gk259*); *sid-1*(*pk3321*)), UA412 (*baIn11*[P_*dat-1*_::α-syn (human, wildtype), P_*dat-1*_::GFP]; *baEx226*[P_*ges-1*_::*mir-2* (pre-miRNA), P_*unc-54*_::tdTomato]; *pgp-8*(*ok2489*)), and UA413 (*baIn11*[P_*dat-1*_::α-syn (human, wildtype), P_*dat-1*_::GFP]; *baEx226*[P_*ges-1*_::*mir-2* (pre-miRNA), P_*unc-54*_::tdTomato]; *pgp-8*(*ok2489*); *sid-1*(*pk3321*)).

### Transgenic line construction

All stable transgenic lines were created by injecting both the transgene and co-injection marker at a concentration of 50 ng/μL. All stable transgenic lines were created by injecting into UA44 worms and then subsequently crossing into indicated mutant backgrounds, except UA272 which was first injected into N2 worms and then subsequently crossed into the UA44 background. A Gateway Entry Clone consisting of the sequence corresponding to the precursor miRNA (pre-miRNA) for *mir-2* with sequence: 5’—TAAACAGTATACAGAAAGCCATCAAAGCGGTGGTTGATGTGTTGCAAATTATGACTTTCATATCACAGCCAGCTTTGATGTGCTGCCTGTTGCACTGT– 3’ was generated using these primers (includes Gateway Tail Sequences):

Forward: 5’ -GGGGACAAGTTTGTACAAAAAAGCAGGCTCCTAAACAGTATACAGAAAGCCATCAAAGC– 3’

Reverse: 5’–GGGGACCACTTTGTACAAGAAAGCTGGGTCACAGTGCAACAGGCAGCACATC– 3’. Gateway Expression Clones including either the *dat-1* or *ges-1* promoter sequence directly upstream of the pre-*mir-2* sequence were generated from this Gateway Entry Clone. Gateway Expression Clones consisting of the *unc-54* promoter sequence directly upstream of the sequences corresponding to either the fluorescent protein tdTomato or mCherry were used as co-injection markers to generate stable transgenic lines.

### DA neurodegeneration analysis

Worms were age synchronized by performing 3-5-hour egg lays. All worms were kept at 20°C for the duration of each experiment. When analyzing stable transgenic lines, great care was taken to isolate and test only worms with the transgenic marker. The extent of neurodegeneration was determined for each replicate by placing worms in a 6 μL drop of 10 mM levamisole (dissolved in 0.5x S Basal buffer) on a glass coverslip. This drop of levamisole on the coverslip was inverted and placed on a 2% agarose pad made on a microscope slide, immobilizing worms to aid in visualization. Using a Nikon Eclipse E600 epifluorescence microscope with the addition of a Nikon Intensilight C-HGFI fluorescent light source, the 6 DA neurons in the head region of worms (4 CEPs and 2 ADEs) were scored for the extent of neurodegeneration by using GFP fluorescence as a proxy. Each worm was scored as either normal or degenerative as previously described [28,82]. Briefly, worms were considered normal only if all 6 DA neurons in the head region had completely intact cell bodies and dendritic processes. Worms were considered degenerative if at least 1 out of the 6 DA neurons was absent, had broken dendritic processes, or exhibited missing cell bodies. Experiments using integrated transgenic lines consisted of 3 biological replicates, and each biological replicate consisted of 30 worms. Experiments using stable transgenic lines without any drug administration consisted of 3 biological replicates (3 separate stable lines), apart from strain UA272 which consisted of 2 biological replicates. Each biological replicate consisted of 1 stable line of each strain, each consisting of 3 technical replicates. Each technical replicate consisted of 30 worms, resulting in a total of 90 worms per biological replicate. AIM-100 experiments using stable transgenic lines consisted of 3 biological replicates (3 separate stable lines). Each biological replicate consisted of 1 stable line of each strain, each consisting of 1 technical replicate. Each technical replicate consisted of 30 worms, resulting in a total of 30 worms per biological replicate. Statistical significance between groups was determined with GraphPad Prism Software.

### Neuron image acquisition

Images of DA neurons were obtained by placing worms in a 6 μL drop of 10 mM levamisole (dissolved in 0.5x S basal Buffer) on a glass coverslip. This drop of levamisole on the coverslip was inverted and placed on a 2% agarose pad made on a microscope slide, immobilizing worms to aid in visualization. Fluorescence microscopy was performed with a Nikon Eclipse E800 epifluorescence microscope equipped with an Endow GFP HYQ filter cube. Images were captured using a Cool Snap CCD camera (Photometrics) with Metamorph software (Molecular Devices).

### Basal Slowing Response (BSR) assays

BSR assays were conducted similarly to as reported by Martinez and colleagues [28]. The BSR assay was performed on day 4 post-hatching and worms were reared on OP50-1 *E*. *coli*. N2 and CB1112 (*cat-2(e1112*)) strains were used to validate the assay: N2 worms exhibit a normal BSR and *cat-2* mutants exhibit a defective BSR. Ring plates (NGM) with a 4 cm outer ring of HB101 *E*. *coli* (OD_600_ of 0.6–0.7 A) and a 1 cm inner circle (unseeded) were used. Individual worms were washed of native bacteria by allowing them to thrash briefly in a 10 μL drop of 0.5 x S basal buffer. Single worms were placed in the center of ring plates in the unseeded portion, upon which time video recording was initiated. Locomotion was recorded using an automated video tracking system (MBF Bioscience) and analyzed using WormLab Software (Version 4.0.5; MBF Bioscience). Videos were taken of individual worms, each on a separate plate. The average peristaltic speed (μm/second) was recorded of the 100 frames before the head of the worm touched the bacterial boundary on the unseeded portion of the ring plate, and the 100 frames after the head touched the bacterial boundary. Three replicates were performed with each strain, each replicate consisting of ten individual worms. The average peristaltic speed on food was compared to the average peristaltic speed off food and converted to a ratio (on/off) that was inverted and normalized to the N2 value to represent BSR as a percentage response compared with N2, which was defined as 100%. Statistical significance between groups was determined with Graphpad Prism Software.

### RNAi Experiments: Plates and bacterial growth conditions

RNAi plates were made by adding ampicillin and Isopropyl β-D-1 thiogalactopyranoside (IPTG) at a final concentration of 100 μg/mL and 1 mM respectively to NGM. RNAi bacteria (HT115 *E*. *coli*) containing either the empty L4440 feeding vector or this same vector containing sequences anti- to target genes desired to be knocked down were grown in LB media with the addition of ampicillin at a final concentration of 100 μg/mL at 37°C while shacking for 16 hours. RNAi bacteria was seeded onto RNAi plates and allowed to fully dry and adhere to the plates in a biological safety cabinet. The resulting plates were then incubated at 20°C overnight to allow for dsRNA induction.

### AIM-100 drug administration

AIM-100 plates were made by first dissolving AIM-100 (Tocris Cat. No. 4946) in a molecular grade 200 proof ethanol stock and then adding to NGM at a final concentration of 100 μM AIM-100 / 0.1% ethanol. Control (solvent) plates contained the same concentration of ethanol (0.1%) as AIM-100 plates, but without the addition of AIM-100. All AIM-100 plates used for experiments were seeded with OP50 *E*. *coli*.

### Rhodamine 123 administration and pixel intensity analysis

Rhodamine 123 plates were made by first dissolving rhodamine 123 (VWR Cat. No. 89139–378) in dimethyl sulfoxide (DMSO) and then adding to NGM at a final concentration of 10 μM. Plates were seeded with OP50 *E*. *coli* after solidification and used for rhodamine 123 treatment immediately. Images of whole worms (N2 and RB1916 (*pgp-8(ok2489)*) 48 hours post-hatching were obtained by placing worms in a 6μL drop of 10 mM levamisole (dissolved in 0.5x S basal Buffer) on a glass coverslip. This drop of levamisole on the coverslip was inverted and placed on a 2% agarose pad made on a microscope slide, immobilizing worms to aid in visualization. Fluorescence microscopy was performed with a Nikon Eclipse E800 epifluorescence microscope equipped with an Endow GFP HYQ filter cube. Images were captured using a Cool Snap CCD camera (Photometrics) with Metamorph software (Molecular Devices). 30 images per replicate were captured per genotype, and each genotype consisted of 3 independent replicates. Once imaged, worms were analyzed for average pixel intensity of rhodamine 123-induced fluorescence using a standardized 50-pixel-diameter circle at the midpoint of the worm bodies using the vulva as an anatomical marker, using the Metamorph software. Representative images of each genotype were taken in the same fashion.

### GFP fluorescence pixel intensity analysis

RNAi plates with either solvent or AIM-100 were made according to the methods outlined in the materials and methods section herein. Images of whole worms expressing GFP in the body wall and pharynx (strain HC46) on day 5 post-hatching were obtained by placing worms in a 6μL drop of 10 mM levamisole (dissolved in 0.5x S basal Buffer) on a glass coverslip. This drop of levamisole on the coverslip was inverted and placed on a 2% agarose pad made on a microscope slide, immobilizing worms to aid in visualization. Fluorescence microscopy was performed with a Nikon Eclipse E800 epifluorescence microscope equipped with an Endow GFP HYQ filter cube. Images were captured using a PCO.PANDA USB3.1 SCMOS Camera with NIS-Elements Software with Clarify.AI. 30 images per replicate were captured per genotype per condition, and each consisted of 3 independent replicates. Once imaged, worms were analyzed for average pixel intensity of GFP fluorescence using a standardized 2048 x 2044-pixel circular region at an anatomically consistent point posterior to the pharynx and anterior to the vulva, using the NIS-Elements Software with Clarify.AI.

### Transcriptomic analysis

RNA was isolated from strains UA44 and UA415 at day 6 post-hatching. 3 separate replicates were isolated for each strain. Large quantities of worms were obtained by performing 2-hour egg lays with 20–30 gravid adults on large NGM plates seeded with OP50-1 *E*. *coli*. All worms destined for RNA isolation were grown at 20°C. Starting at day 4 post-hatching, 400 worms were transferred to 100 mm fresh seeded plates to prevent starvation and mixing of target worms and progeny. At day 6 post-hatching, for each strain, for each replicate, 380 worms were transferred to an unseeded NGM plate. Great care was taken to only transfer target worms and to avoid carryover of embryos. Worms were washed off unseeded plates with 0.5x M9 into sterilized glass conical tubes. Worms were washed 4 times with 0.5x M9 to reduce bacteria in samples. After the 4^th^ wash, worms were purged for 20 minutes to reduce the number of bacteria in the gut of the worms, and this was done by rocking the tubes back and forth on a nutator. Worms were then washed 2 times with double-distilled deionized water to rid of any remaining bacteria. Worms were then transferred to 1.5 mL RNAse-free lo-bind microcentrifuge tubes and as much supernatant was removed as possible. 500 μL of Trizol (ThermoFisher Scientific) was then added to each tube, and then immediately vortexed for 30 seconds and placed in liquid nitrogen until frozen completely. Tubes were then thawed at 37°C. This freeze/thaw process was then performed the same way 6 more times. After the last thaw, all tubes were vortexed for 30 seconds and then put on ice for 30 seconds. This vortex/ice process was then performed the same way 7 more times. All tubes were then allowed to incubate at room temperature for 5 minutes. Then 100 μL of chloroform was added to each tube, and each tube was immediately inverted continuously for 15 seconds. The tubes were then allowed to incubate at room temperature for 5 minutes to allow for phase separation. The tubes were then centrifuged for 15 minutes at 4°C at 12,000 rpm. The top aqueous phase from each tube was then transferred to new 1.5 mL RNAse-free microcentrifuge tubes, and an equal volume of RNAse-free 70% ethanol was added to each tube and mixed by gentle inversion. At this point, the aqueous phase mixed with ethanol was subjected to the protocol outlined in the Qiagen RNeasy Micro Kit, and reagents and materials from this kit were used for the remainder of the isolation. Ultimately, RNA was eluted with 35 μL of RNAse-free water.

RNA samples were sent to the Novogene Corporation, Inc.; Sacramento, CA. Novogene performed eukaryotic mRNA sequencing as well as bioinformatic analysis to determine DEGs, transcript levels, log2fold changes, and adjusted and non-adjusted p-values. An Illumina HiSeq platform was used for a paired-end 150 basepair sequencing strategy (short reads) to sequence cDNA libraries corresponding to RNA samples. Transcript expression levels were determined using the RPKM (Reads Per Kilobases per Million reads) method [83]. Data was filtered by removing adaptors and low-quality reads. Bioinformatic analysis was done using HISAT2, version 2.1.0-beta [84], with a parameter of “mismatch = 2” to map samples to the N2 reference genome (ftp://ftp.ebi.ac.uk/pub/databases/wormbase/parasite/releases/WBPS13/species/caenorhabditis_elegans/PRJNA13758/caenorhabditis_elegans.PRJNA13758.WBPS13.genomic.fa.gz). The resultant BAM files from HISAT2 were input for quantification of reads with HTSeq, version 0.6.1, with parameter “-m union” [85]. Differentially expressed genes (DEGs) were extracted using DESeq version 1.10.1 [86]. Significance was determined if the p-adjusted value was less than 0.05. DESeq utilized a Benjamini-Hochberg method of normalization to calculate the adjusted p-value, a powerful method for controlling the false discovery rate. Heatmaps were created in R using package “pheatmap” (Kolde, 2015). Gene ontology (GO) analyses were created using the WormCat bioinformatics resource [87]. Genes that were significantly up- or down-regulated as determined by an adjusted p-value of less 0.05 were used in the analysis.

### Real-time Quantitative PCR

RNA was isolated from worms and RT-qPCR was performed as described previously [88]. Briefly, total RNA was isolated from 100 young adult (α-syn + Solvent, α-syn + AIM-100: day 4 post-hatching; α-syn + SID mutants: day 10 post-hatching) animals from each group using TRI reagent (Molecular Research Center). Samples were rid of genomic DNA contamination with 1 μl of DNaseI (Promega) treatment for 60 minutes at 37°C, then with DNase Stop solution for 10 minutes at 65°C. 1μg of RNA was used for cDNA synthesis using the iScript Reverse Transcription Supermix for RT-qPCR (Bio-Rad) following the manufacturer’s protocol. RT-qPCR was performed using IQ-SYBR Green Supermix (Bio-Rad) with the Bio-Rad CFX96 Real-Time System. Reactions consisted of 7.5 μl of IQ SYBR Green Supermix, 200 nM of forward and reverse primers, and 5 ng of cDNA, to a final volume of 15 μl. The thermocycling conditions were as follows: polymerase activation and DNA denaturation at 95°C for 3 minutes, then 35 cycles of 10 seconds at 95°C, 30 seconds at 60°C., A melting curve analysis was performed after the final cycle using the default setting of CFX96 Real-Time System. Single melt peaks were observed for each targeted gene. The PCR efficiency for each primer pair was calculated from standard curves generated using serial dilutions: Eα-syn = 99.0%, E*snb-1* = 99.8%, E*tba-1* = 99.5%, E*ama-1* = 103.1%. The expression levels of α-syn were normalized to three reference genes: *snb-1*, *tba-1*, and *ama-1*. NTC and NRT controls exhibited no amplification. All reference genes used were analyzed by GeNorm and passed for target stability. All strains tested consisted of 3 independent biological replicates, each consisting of 3 technical replicates, for each target gene tested. Data analysis was executed by the Gene Expression Module of CFX Manager software.

The following primers were used for the assays:

α-syn Forward: ATGTAGGCTCCAAAACCAAGG

α-syn Reverse: ACTGCTCCTCCAACATTTGTC

*snb-1* Forward: CCGGATAAGACCATCTTGACG

*snb-1* Reverse: GACGACTTCATCAACCTGAGC

*tba-1* Forward: ATCTCTGCTGACAAGGCTTAC

*tba-1* Reverse: GTACAAGAGGCAAACAGCCAT

*ama-1* Forward: TCCTACGATGTATCGAGGCAA

*ama-1* Reverse: CTCCCTCCGGTGTAATAATGA

### Statistical analysis

All statistical analysis was performed with GraphPad Prism Software (Version 9.0.0).

When analyses were performed with data containing multiple days, comparing every group to every other group, a Two-Way ANOVA was used with Tukey’s *post hoc* analysis. When analyses were performed with data containing multiple days, comparing only groups within the same day to each other, a Two-Way ANOVA was used with a Šídák’s *post hoc* analysis. When analyses were performed with data containing one single day, and there were only two groups, an unpaired, two-tailed t-test was used. When analyses were performed with data containing one single day, with more than two groups and only comparing back to a control, a One-Way ANOVA was used with Dunnett’s *post hoc* analysis. All data assessed involving AIM-100 administration used a Two-Way ANOVA with Tukey’s *post hoc* analysis.

## Supporting information

S1 Table Strain list. List of *C*. *elegans* strains used in this study.
(DOCX)

S2 Table Values for all experiments. A spreadsheet inclusive of all data collected for the experiments; each tab displays the data from a single graph.
(XLSX)
