# Supplementary material for: Systemic RNA Interference Defective (SID) genes modulate dopaminergic neurodegeneration in C. elegans
Source: PLoS Genet. 2022 Aug 19;18(8):e1010115. doi: 10.1371/journal.pgen.1010115 (PMC9432717; doi:10.1371/journal.pgen.1010115)
Supplement: S1 Table — List of C. elegans strains used in this study. (DOCX) [file pgen.1010115.s001.docx]

Strain Genotype Source References

N2 wildtype (Bristol) CGC

CB1112 *cat-2(e1112)*  CGC

RB1916 *pgp-8(ok2489)* CGC

NL3321 *sid-1(pk3321)* CGC

VC787 *sid-3(ok973)*  CGC

VC495 *mir-2(gk259)*  CGC

HC46 *ccIs4251* [P_myo_*_-_*_3_::GFP-NLS*,*P_myo-3_::GFP-MITO ] I;

*mIs11*[P_myo-2_::GFP ] IV  Hunter lab [1]

BY250 *vtIs7* [P_dat-1_::GFP] Blakely lab [2]

UA44 *baIn11*[P_dat-1_::α-syn, P_dat-1_::GFP] Caldwell lab [3]

UA196 *sid-1(pk3321)*; *baIn33*[P_dat-1_::*sid-1*, P_myo-2_::mCherry]; " [4]

*baIn11*[P_dat-1_::α-syn, P_dat-1_::GFP]

UA273 *baEx161*[P_dat-1_::*mir-2*, P_unc-54_::mCherry]; " this paper

*baIn11*[P_dat-1_::α-syn, P_dat-1_::GFP]

UA372 *baIn54*[P_dat-1_::A53T α-syn, P_unc-54_::tdTomato] " "

UA408 *baEx226*[P_ges-1_::*mir-2* short, P_unc-54_::tdTomato]; " "

*baIn11*[P_dat-1_::α-syn, P_dat-1_::GFP]

UA409 *baEx226*[P_ges-1_::*mir-2* short, P_unc-54_::tdTomato]; " "

*baIn11*[P_dat-1_::α-syn, P_dat-1_::GFP]; *mir-2(gk259)*

UA410 *baEx226*[P_ges-1_::mir-2 short, P_unc-54_::tdTomato]; " "

*baIn11*[P_dat-1_::α-syn, P_dat-1_::GFP]; *sid-1(pk3321)*

UA411 *baEx226*[P_ges-1_::*mir-2* short, P_unc-54_::tdTomato]; " "

*baIn11*[P_dat-1_::α-syn, P_dat-1_::GFP]; *mir-2(gk259); sid-1(pk3321)*

UA412 *baEx226*[P_ges-1_::*mir-2* short, P_unc-54_::tdTomato]; " "

*baIn11*[P_dat-1_::α-syn, P_dat-1_::GFP]; *pgp-8(ok2489)*

UA413 *baEx226*[P_ges-1_::*mir-2* short, P_unc-54_::tdTomato]; " "

*baIn11*[P_dat-1_::α-syn, P_dat-1_::GFP]; pgp-8(ok2489); sid-1(pk3321)

UA414 *sid-1(pk3321); sid-3(ok973); baIn11*[P_dat-1_::α-syn, P_dat-1_::GFP] " "

UA415 *sid-1(pk3321), baIn11*[P_dat-1_::α-syn, P_dat-1_::GFP] " "

UA416 *sid-3(pk973), baIn11*[P_dat-1_::α-syn, P_dat-1_::GFP] " "

UA417 *mir-2(gk259); sid-1(pk3321); baIn11*[P_dat-1_::α-syn, P_dat-1_::GFP] " "

UA418 *mir-2(gk259); baIn11*[P_dat-1_::α-syn, P_dat-1_::GFP] " "

UA419 *pgp-8(ok2489); baIn11*[P_dat-1_::α-syn, P_dat-1_::GFP] " "

UA420 *baIn54*[P_dat-1_::A53T α-syn, P_unc-54_::tdTomato]; *sid-1(pk3321)* " "

UA421 *baIn54*[P_dat-1_::A53T α-syn, P_unc-54_::tdTomato]; *sid-3(ok973)*  " "

UA423 *sid-1(pk3321), vtIs7*[P_dat-1_::GFP] " "

UA436 *pgp-8(ok2489); sid-1(pk3321); baIn11*[P_dat-1_::α-syn, P_dat-1_::GFP] " "

UA437 *mir-2(gk259); sid-1(pk3321); baIn33*[P_dat-1_::*sid-1*, " "

P_myo-2_::mCherry]; *baIn11*[P_dat-1_::GFP,P_dat-1_::α-syn]

UA444 *mir-251*(n4606); *baIn11*[P_dat-1_::α-syn, P_dat-1_::GFP] " "

UA445 *mir-249(n4983)*; *baIn11*[P_dat-1_::α-syn, P_dat-1_::GFP] " "

UA446 *mir-360(n4635);* *baIn11*[P_dat-1_::α-syn, P_dat-1_::GFP] " "

**References**

1. Winston WM, Melodowitch C, Hunter CP. Systemic RNAi in *C. elegans* Requires the Putative Transmembrane Protein SID-1. Science. 2002; *295*, 2456-2459. doi: 10.1126/science.1068836.
2. Nass R, Hahn MK, Jessen T, McDonald PW, Carvelli L, Blakely RD. A genetic screen in *Caenorhabditis elegans* for dopamine neuron insensitivity to 6-hydroxydopamine identifies dopamine transporter mutants impacting transporter biosynthesis and trafficking. J Neurochem. 2005; *94*, 774-85. doi: 10.1111/j.1471-4159.2005.03205.x.
3. Pivtoraiko VN, Harrington AJ, Mader BJ, Luker AM, Caldwell GA, Caldwell KA, Roth KA, Shacka JJ. Low-dose bafilomycin attenuates neuronal cell death associated with autophagy-lysosome pathway dysfunction. J Neurochem. 2010; *114*,1193-204. doi: 10.1111/j.1471-4159.2010.06838.x.
4. Harrington AJ, Yacoubian TA, Slone SR, Caldwell KA, Caldwell, GA. Functional analysis of VPS41-mediated neuroprotection in *Caenorhabditis elegans* and mammalian models of Parkinson’s disease. J. Neurosci. 2012; *32*, 2142-2153. doi: 10.1523/JNEUROSCI.2606-11.2012.
